# Synthesis of 3,4-dihydropyrimidines and octahydroquinazolinones by SBA-15 supported schiff-base iron (III) complex as durable and reusable catalyst under ultrasound irradiation

**DOI:** 10.1038/s41598-024-65519-x

**Published:** 2024-06-27

**Authors:** Zeynab Balali, Javad Safaei-Ghomi, Elahe Mashhadi

**Affiliations:** Department of Organic Chemistry, Faculty of Chemistry, University of Kashan, Kashan, I. R. of Iran

**Keywords:** Synthetic chemistry methodology, Heterogeneous catalysis

## Abstract

Biginelli-type heterocyclic compounds are particularly important due to their several chemical reactivities and various range of pharmacological activity. Therefore Biginelli reaction has witnessed several modification and numerous investigations are continuing in this field to develop more effective and efficient methodologies. In this research, Iron (III) schiff base immobilized SBA-15 has been prepared as a valuable, efficient, and recoverable catalyst for the Biginelli reaction. The morphology of the prepared catalyst was identified by spectroscopic characterization techniques and structural microscopic analysis including Fourier transform infrared (FT-IR) patterns, X-ray diffraction (XRD) by powder crystal method, Energy-dispersive X-ray spectroscopy (EDS) study, Thermogravimetric-Differential thermal analysis (TGA-DTA), Transmission electron microscopy (TEM) and Field emission scanning electron microscopy (FE-SEM) images. Biginelli compounds containing 3,4-dihydropyrimidines and octahydroquinazolinones were conveniently synthesized by this catalyzed protocol from the cycloaddition of aromatic aldehydes with the 1,3-dicarbonyl substrates and urea via ultrasonic waves. The several advantages of the presented approach are high yields and easy isolation of products, shorter reaction times, and milder conditions, structural stability and reusable catalyst. The combination of heterogeneous catalyst and ultrasonic radiation can make catalytic reactions more efficient than traditional ways attractive for academic researchers and application scholars in the industry.

## Introduction

**Scheme 1:**
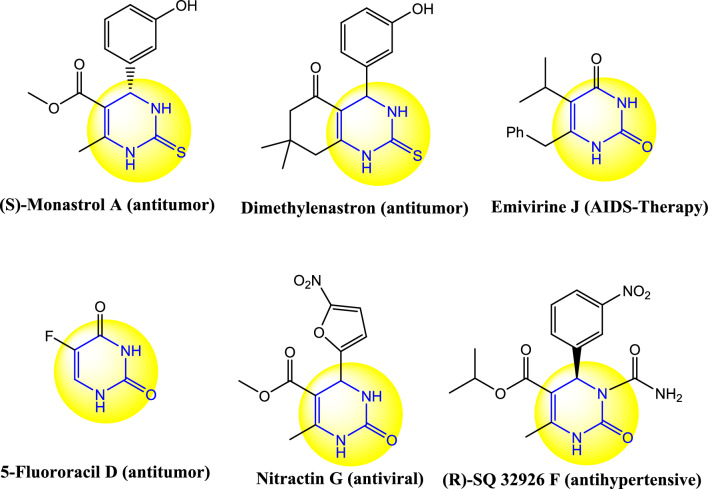
Examples of biological activities of dihydropyrimidinon heterocycles.

In medicinal and organic synthetic techniques, preparation of 3,4-dihydropyrimidin-2(1*H*)-one compounds as biological active molecules have great importance among other promising categories. Some of the biological properties of the dihydropyrimidinone scaffolds include antiulcer, antiviral, antimicrobial, anticancer, antioxidant, antiparasitic, antihypertensive, and calcium channel modulation^1,2^. Representative examples of biologically active dihydropyrimidinone derivatives are shown in Scheme 1.

Dihydropyrimidinone heterocycles were first synthesized by the Italian chemist Pietro Biginelli in 1893^3^. Therefore, these heterocycles are known as Biginelli compounds. The dihydropyrimidinone rings are prepared by condensation of numerous aldehydes with (thio)urea and 1,3-dicarbonyl compounds such as *β*-ketoester and cyclic *β*-diketone substrates. Subsequently, this organic transformation was conducted by various catalytic systems such as Fe_3_O_4_ supported hydrogen sulfate ionic liquid^4^, FeCl_3_^5^, sulfamic acid pyromellitic diamide-functionalized MCM-41^6^, cis–[Mo^VI^O_2_] complexes^7^, Cu-Schiff base-MCM-41^8^, ZrOCl_2_⋅8H_2_O^9^, CuCl_2_ 2H_2_O^10^, bis(p-sulfoanilino)triazine-functionalized silica-coated magnetite nanoparticles^11^, Nano-Fe_3_O_4_-bpy-Ni(II) ^12^, bentonite/PS-SO_3_H^13^, copper^14^, among others.

Nowadays, the transition metal complexes with various ligands have attracted great attention as active catalytic systems for a wide range of reactions. The complex ligands are used to bond to the metal ion through a pair of donor atoms or a donor atom and a lone electron pair^15,16^. Due to the remarkable development of transition metal–ligand complexes as heterogeneous catalysts, suitable supports were applied such as Boehmite nanoparticles^17^, metal–organic frameworks (MOFs)^18^, magnetic nanoparticles^19^, types of silica^20^ and polymers^21^.

Additionally, several research papers reported that supported Schiff base metal complexes were applied in heterogeneous catalysis. Different types of supports were used for the stabilization of Schiff base metal complexes including MOF^22^, silica^23^, Fe_3_O_4_ nanoparticles^24^, graphene oxide^25^, carbon nanotube^26^, HY zeolite^27^, and SBA-15 mesoporous silica^28^. Among the support materials, SBA-15 mesoporous silica is one of the valuable and useful supports for the transition metal–ligand complexes with outstanding advantages of high surface area, large pore diameters (with an overall sizing range of 3–30 nm with narrow size distributions), high stable structure, biocompatibility, easy functionalization, and convenient catalyst recycling. It should be mentioned that the covalent bonds formed between the SBA-15 silica and the Schiff base metal complexes, which results in a catalyst with more resistance to leaching during the catalytic reaction^28,29^.

**Scheme 2:**
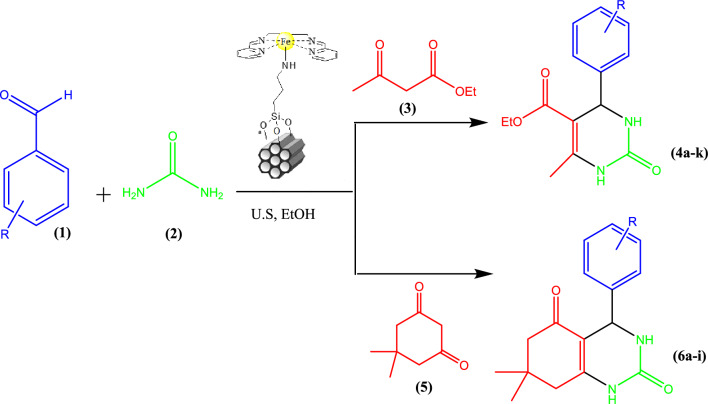
Modified sonocatalyzed Biginelli-type reaction using SBA-15@Schiff-base@Fe(III).

On the other hand, the application of ultrasonic energy and heterogeneous catalysts in a chemical process has been useful in enhancing the catalytic reaction rates (improvement of heat and mass transfer), accelerating dissolution, reducing energy consumption, and renewing the surface of a solid catalyst or reactant in a variety of reaction systems^30^. Therefore, in continuation of the studies of our research group for the design of heterogeneous catalysts for organic chemical processes^31,32^, we have developed the classical Biginelli-type reaction to prepare 3,4-dihydropyrimidines and octahydroquinazolinones using SBA-15@Schiff-base@Fe(III) as heterogeneous and reusable catalyst (Scheme 2).

## Experimental section

### Structural and morphological characterizations of the catalyst SBA-15@Schiff-base@Fe(III)

**Scheme 3:**
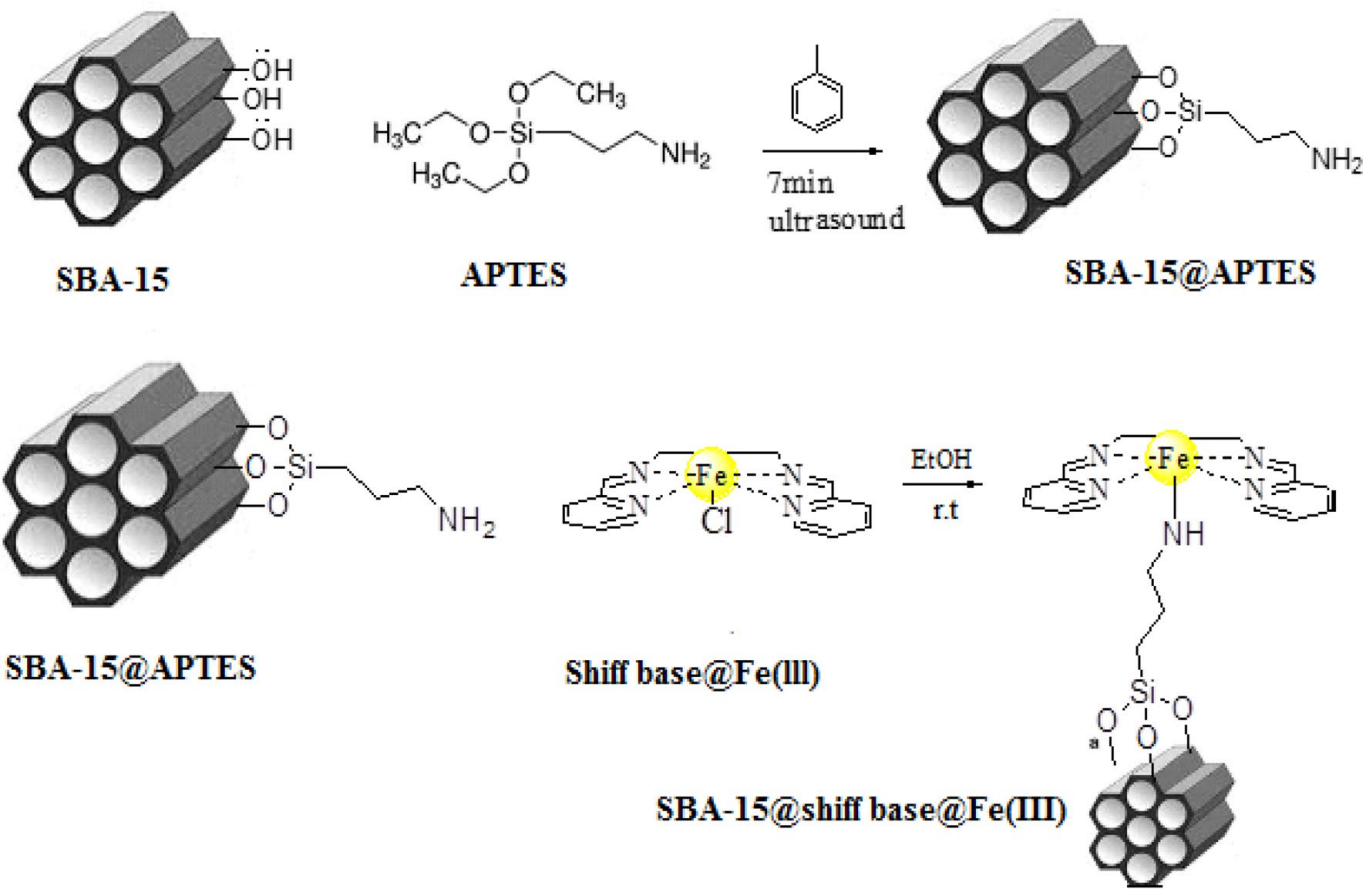
The synthesis of the catalyst SBA-15@Schiff‐base@Fe(III).

According to the synthetic route shown in Scheme 3, Schiff‐base@Fe(III) complex immobilized on the SBA-15 was synthesized in three stages. First, SBA-15 was prepared according to the mentioned procedure in literature^33^. Then, SBA-15@APTES was synthesized by employing 3-aminopropyltriethoxysilane (APTES) in toluene solvent under ultrasound irradiation^34^. On the other hand, Schiff base was synthesized by the reaction of ethylenediamine with pyridine-2-carbalde in EtOH under reflux conditions^23^. After that, Schiff‐base@Fe(III) was prepared via the reaction of FeCl_3_.6H_2_O and *N,N′‐*bis‐(pyridin‐2‐ylmethylene)‐ethane‐1,2‐diamine in EtOH under reflux condition. In the next step, The anchoring of Schiff‐base@Fe(III) complex onto SBA‑15@APTES was formed through the chemical reaction between free amine groups anchored on SBA-15 and Cl ion of Shiff-base@Fe(III) to obtain an efficient heterogeneous catalyst^35^.

**Figure 1:**
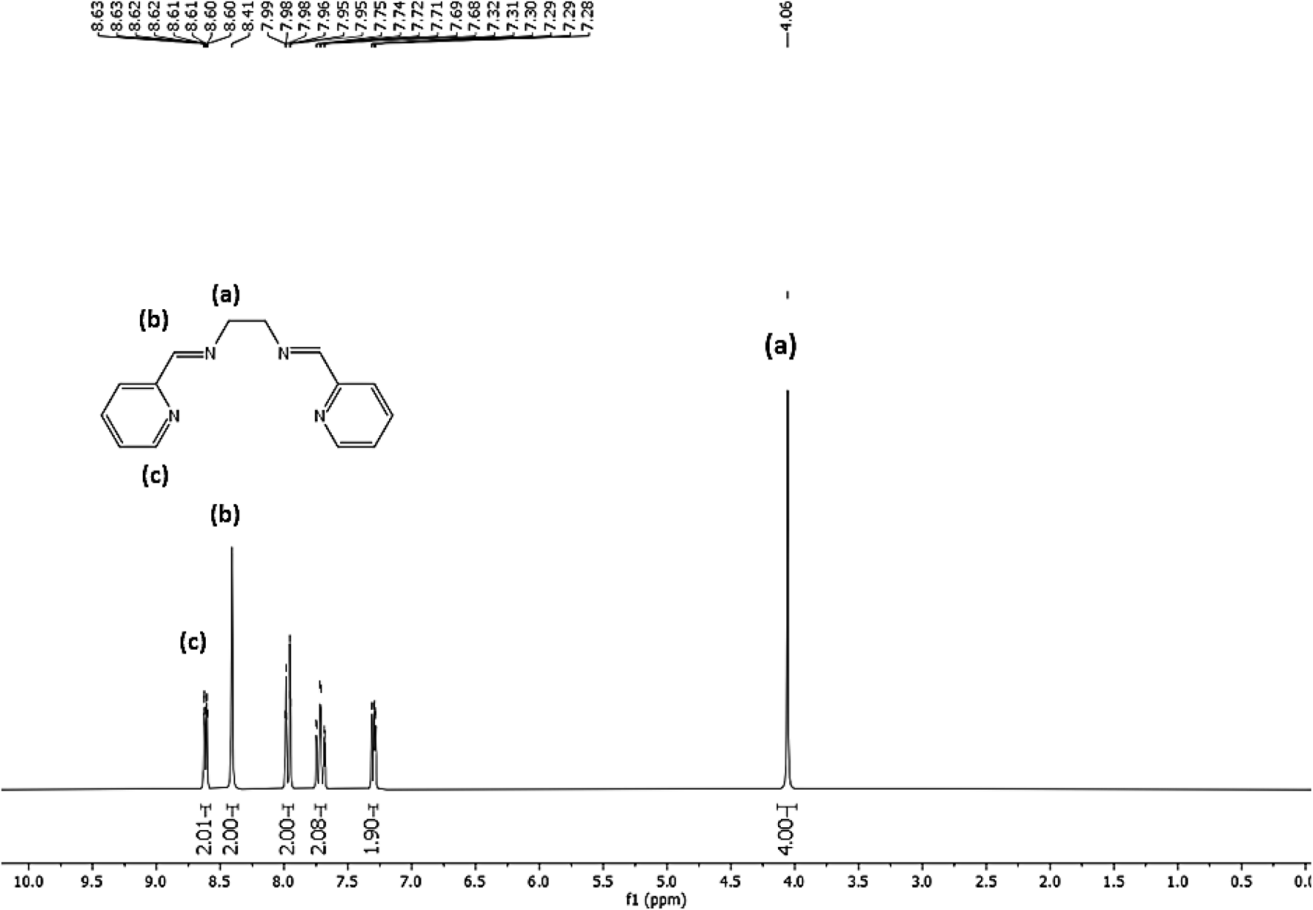
^1^H-NMR spectra of *N,N'*-bis-(pyridin-2-ylmethylene)-ethane-1,2-diamine (Schiff-base).

^1^H NMR spectroscopy was utilized to confirm the successful creation of *N,N'*-bis-(pyridin-2-ylmethylene)-ethane-1,2-diamine (Schiff base), as demonstrated in Fig. 1. Schiff base shows five types of protons in the aromatic region, where CH protons adjacent to pyridyl nitrogen (c) appear at δ = 8.61 ppm as a doublet of doublet of doublet, while azomethine protons (–CH=N–) as a singlet (b) appear at δ = 8.41 ppm. Moreover, the peak at δ = 4.06 ppm is related to CH_2_ protons (a) in the aliphatic region^35^.

**Figure 2:**
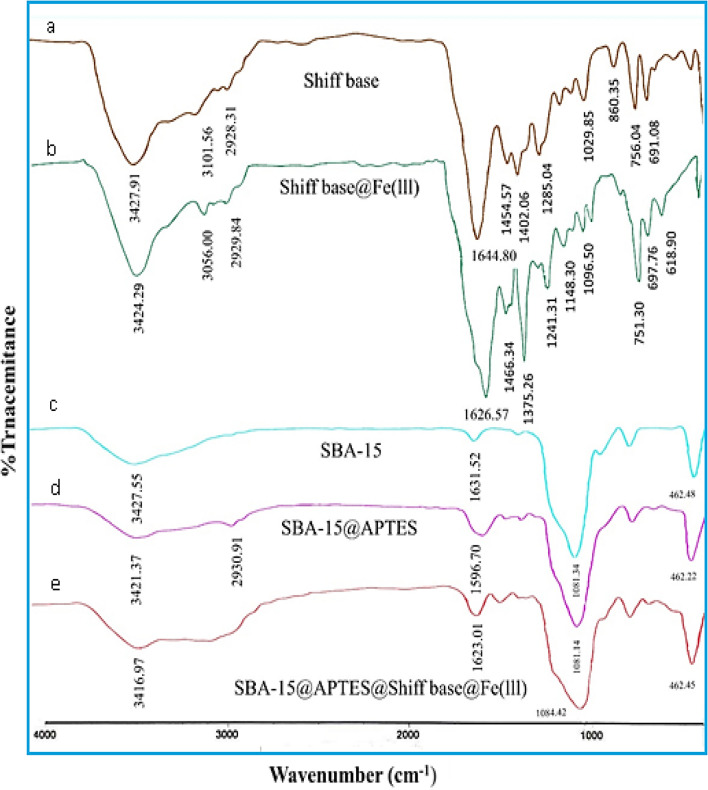
FT-IR spectra of Schiff-base (a), Schiff-base@Fe(III) (b), SBA-15 (c), SBA-15@APTES (d) and SBA-15@Schiff‐base@Fe(III) (e).

The FT-IR spectra of synthesized materials are shown in Fig. 2. The characteristic strong peak at 1644 cm^−1^ represents the stretching of C=N bands of Schiff-base. This band shifted to around 1626 cm^−1^ in the spectrum of the Schiff‐base@Fe(III) complex indicateing the coordination of the azomethine nitrogen to the Fe(III) ions. In the SBA-15 silica framework, the predominant peaks at 3427 cm^–1^ and 1081 cm^–1^ are attributed to the stretching vibrations of Si–OH and Si–O-Si bonds, respectively^34^. After silica modification with the propyl amine groups, a new multiple peak appeared at around 2931 cm^−1^ due to aliphatic CH_2_ stretching bonds in SBA@APTES. Moreover, the characteristic bands of –NH_2_ at 3421 and 1596 cm^−1^ show that the link between the SBA-15 and the APTES is done. The FT-IR spectrum of SBA-15@Schiff‐base@Fe(III) shows not only the pure SBA-15 bands including the peaks between 1000 and 1200 cm^−1^, which is due to stretching frequencies of (Si–O–Si) bonds, but also it shows the C=N stretching band of the synthesized complex at about 1623 cm^−1^. This peak can be attributed to the presence of the Schiff‐base@Fe(III) groups in the mesoporous silica framework^35^.

**Figure 3:**
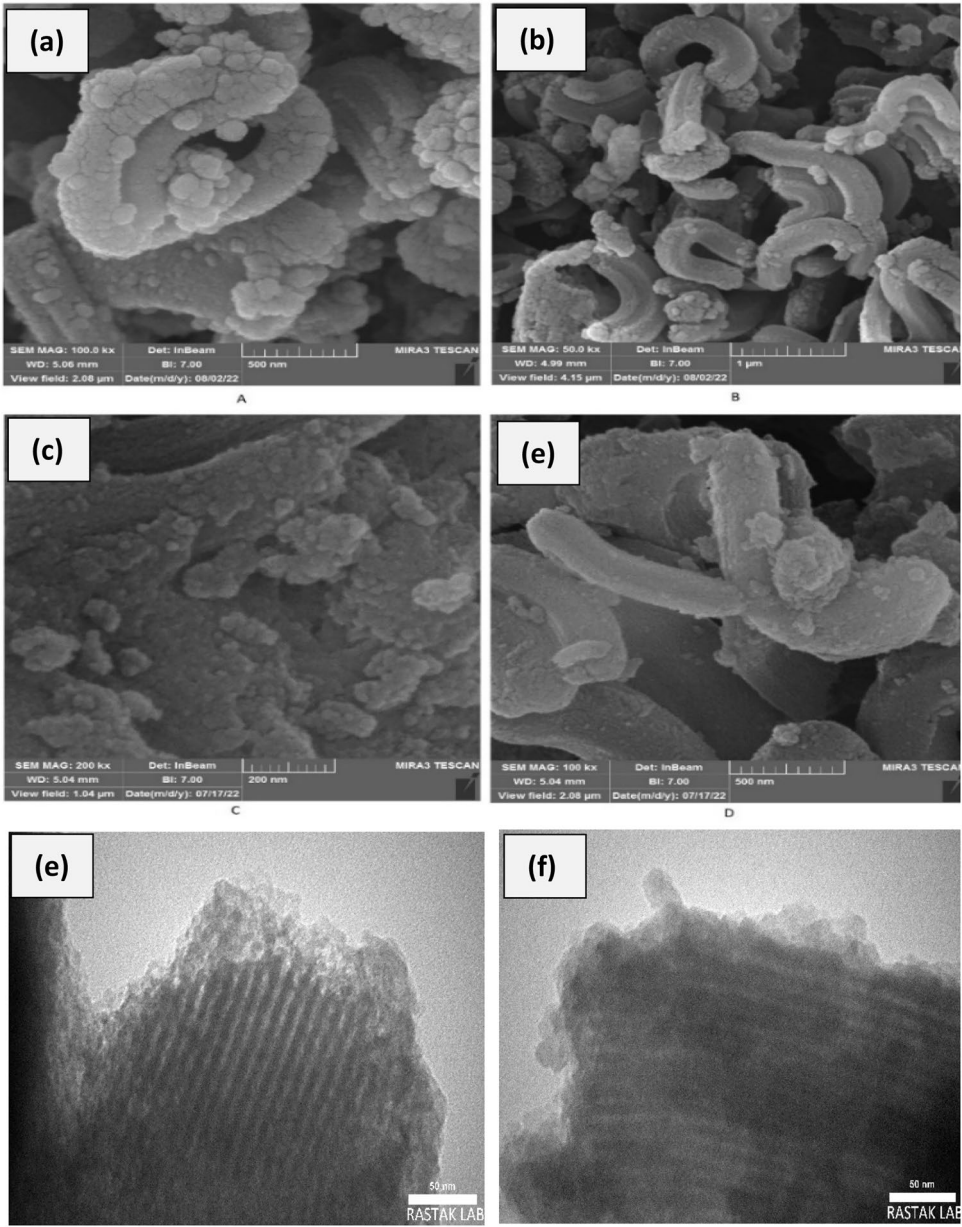
SEM images of SBA-15 (**a** and **b**), SBA-15@Schiff‐base@Fe(III) (**c** and **d**) and TEM images of SBA-15@Schiff‐base@Fe(III) (**e** and **f**).

To obtain the structure of surface morphology of the pure SBA-15 and SBA-15@Schiff‐base@Fe(III), SEM images of the silica mesoporous were obtained. As shown in the Fig. 3a and b, the SBA-15 sample has the two-dimensional hexagonal structure with relatively uniform sizes. After complex formulation, the shape of SBA-15@Schiff‐base@Fe(III) is unchanged noticeably (Fig. 3c and d). The TEM image of the SBA-15@Schiff‐base@Fe(III) sample (Fig. 3e and f) reveals that iron cannot be observed in the silica pores, which shows coordination of the Fe^3+^ ions with N atoms in Schiff‐base ligand is stable after immobilization process^34^.

**Figure 4:**
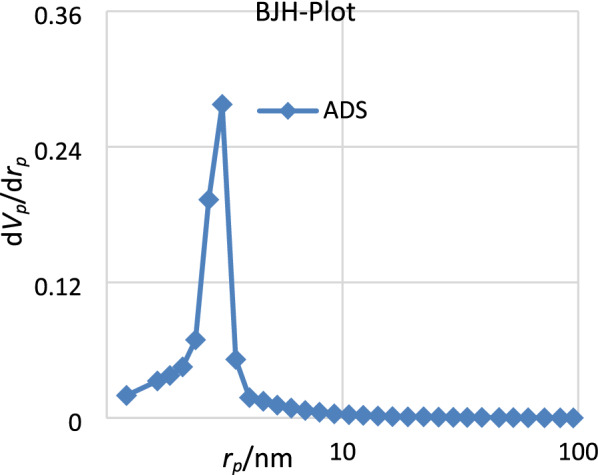
The BET of SBA-15@Schiff‐base@Fe(III).

The BET surface area was observed for the supported complex in Fig. 4. The Estimated pore volume and pore dimension of SBA-15@Schiff‐base@Fe(III) are found 0.36 cm^3^ g^−1^ and 7.6 nm, respectively.

**Figure 5:**
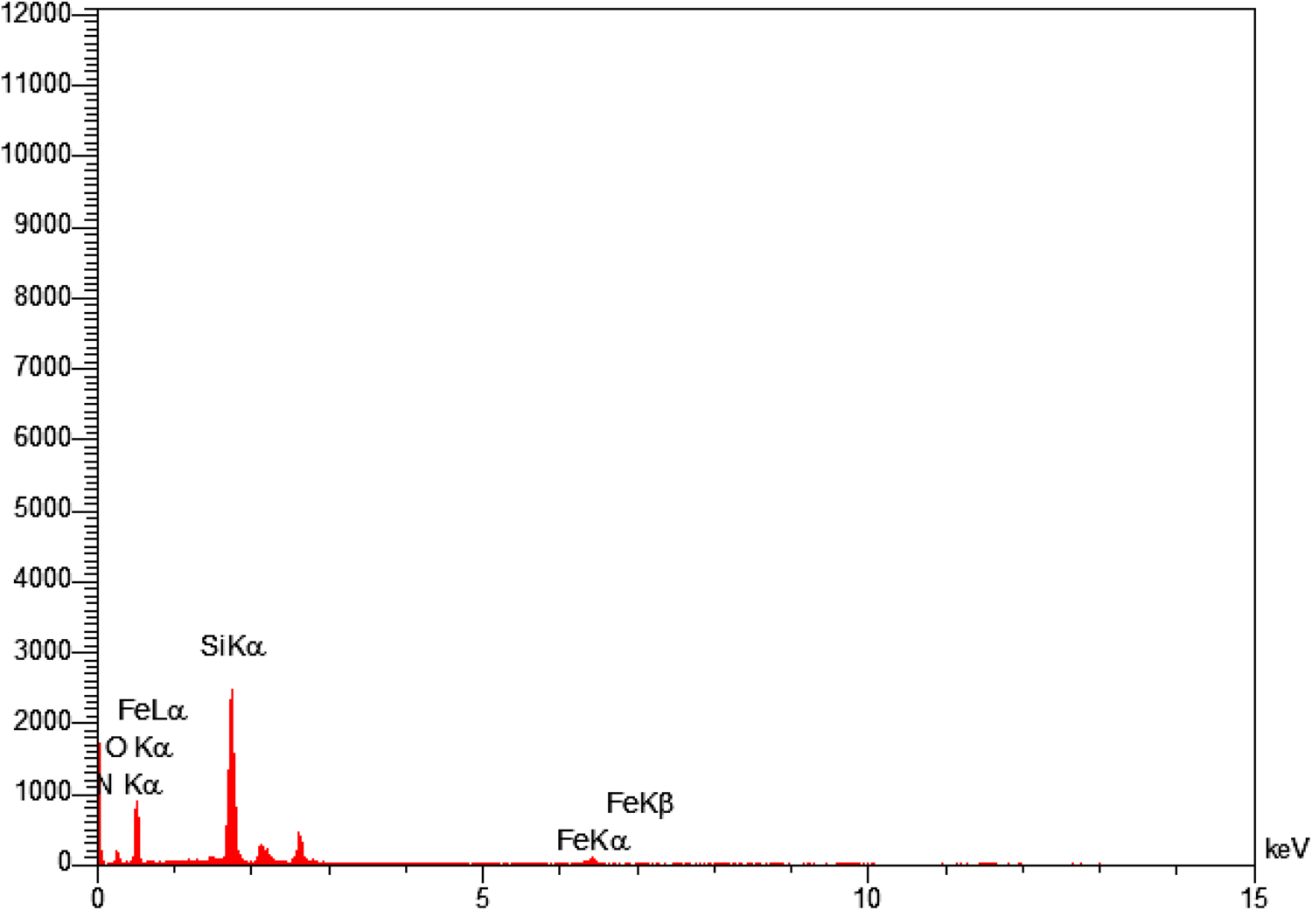
Energy-dispersive X-ray spectroscopy of SBA-15@Schiff‐base@Fe(III).

**Figure 6:**
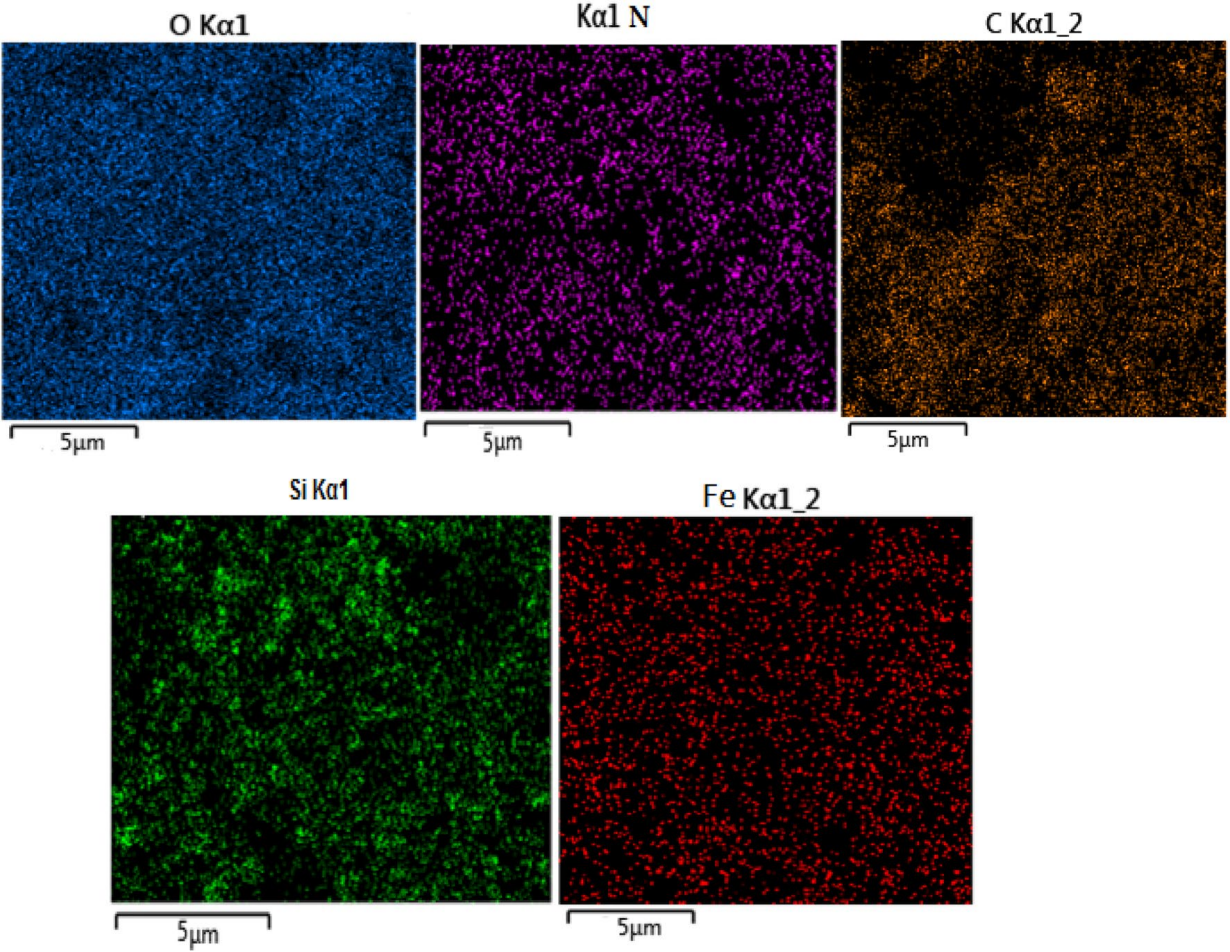
The elemental mapping of SBA-15@Schiff‐base@Fe(III).

The obtained result of the EDX analysis is shown in Fig. 5. The elemental composition of SBA-15@Schiff‐base@Fe(III) confirms the presence of iron, nitrogen, oxygen, and silicon elements with 2.63, 3.02, 39.77, and 54.58 (wt%) respectively, in the modified silica. The elemental mapping images conducted on a selected segment of the Schiff-base@Fe(III) supported on SBA-15 revealed a consistent and homogeneous distribution of these elements (Fig. 6).

**Figure 7:**
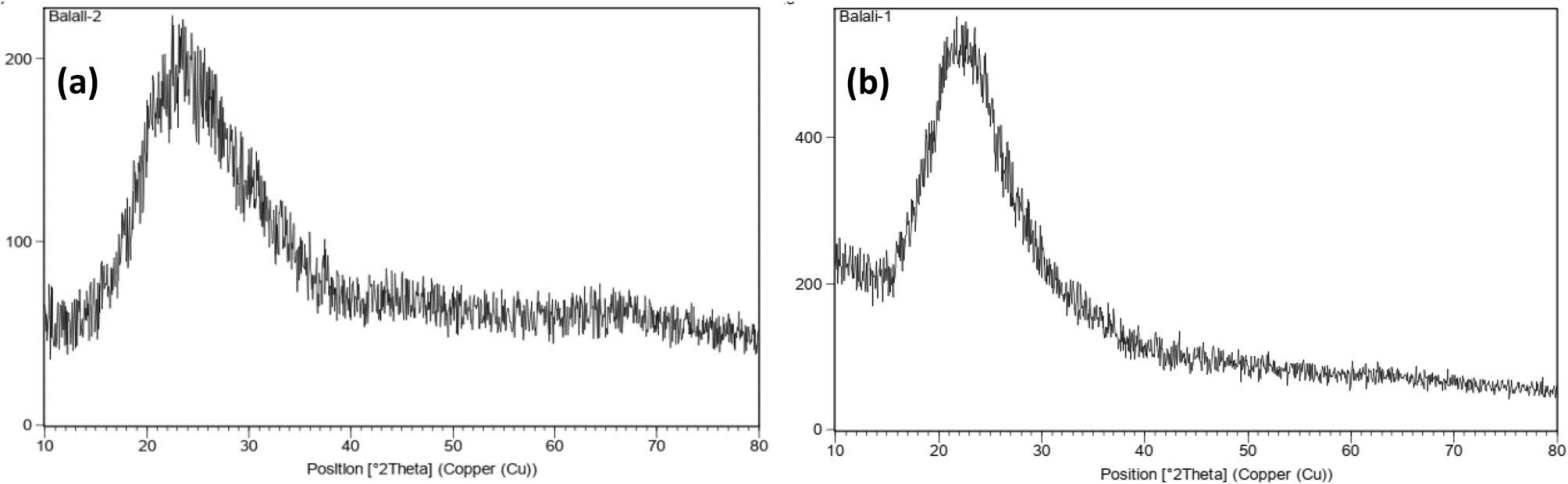
The XRD patterns for Pure siliceous SBA-15 (**a**) and SBA-15@Schiff‐base@Fe(III) (**b**).

The XRD peaks of unmodified SBA-15 and SBA-15@Schiff‐base@Fe(III) are shown in Fig. 7. The sample of SBA-15 exhibited only a broad peak at *2θ* = 22.9° as the (100) reflection, which is a characteristic signal of the amorphous nature of silica^36^. The other two weak diffraction peaks (210) and (110) are not clear, suggesting that the ordered channels of SBA-15 were not prominent^37^. Similar results were obtained for SBA-15@Schiff‐base@Fe(III). This case can be explained that Schiff‐base@Fe(III) is highly dispersed through SBA-15@APTES framework because of the absence of any crystalline phase after immobilization, and only Schiff‐base@Fe(III) molecules coordinated to the isolated NH_2_ groups are present in the modified SBA-15. However, for SBA-15@Schiff‐base@Fe(III), their intensity was slightly reduced, indicating a slight, decrease in the mesoporous structure due to the blocking of holes during the Schiff‐base ligand addition^38^.

**Figure 8:**
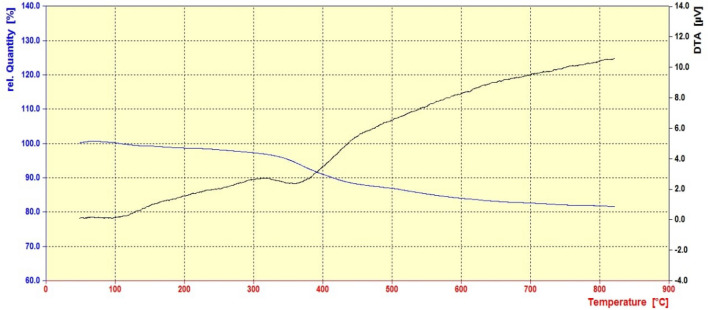
The TGA-DTA diagram of Schiff-base@Fe(III) supported on SBA-15.

Figure 8 illustrates the TGA-DTA of the Schiff-base@Fe(III) supported on SBA-15. It shows a mass loss between 200 and 700 °C, which could be interpreted to loss of organic spacer and Schiff-base complex^29^. DTA diagram indicates that decomposition of modified silica is an exothermic process. Moreover, the obtained results indicate that the Schiff-base@Fe(III) anchored SBA-15 exhibits impressive thermal stability up to 600 °C.

Biginelli reaction is a practical and interesting manner to chemists researchers due to the importance of these heterocycles in medicinal chemistry. Therefore, the catalytic activity of SBA-15@Schiff‐base@Fe(III) has been investigated in the Biginelli condensation for synthesis of dihydropyrimidinone and octahydroquinazolinone derivatives. To optimize this chemical reaction conditions, we carried out the condensation of the organic compounds including benzaldehyde (1 mmol), ethyl acetoacetate (1 mmol), and urea (1.5 mmol) in EtOH solvent as the standard reaction. As shown in Table 1, satisfactory results of reaction products were obtained by 0.03 g of the catalyst under reflux conditions with 85% yield of the desired product (Entry 10). This reaction produced no product without a catalyst (Entry 1). We tried to improve the reaction conditions and product efficiency with ultrasonic energy. The results show that the amount of the Biginelli product is almost low (53%) under catalyst-free conditions via ultrasonic strategy (Entry 11). But, the highest yield (˃95%) was obtained in the presence of the catalyst under ultrasonic irradiation (Entry 14). Ultrasonic waves create cavitation bubbles on the surface of the heterogeneous catalyst. In fact, the solid catalyst acts as a nucleus for the formation of cavitation bubbles in the sonication procedure. The implosion of cavitation bubbles on the surface of the catalyst provides locally enough energy to improve of the reaction rate^30^. In order to evaluate the optimized reaction medium, various solvents including ethanol, H_2_O, acetonitrile, THF, and DMSO were examined by sonication (Entries 16–19). Catalytic cycloaddition took place in ethanol as the most efficient solvent system. The results could be explained by the much better solubility of the organic molecules and the much better dispersion of the catalyst in the EtOH solvent.

**Table 1:** Optimized conditions to synthesize of 3,4-dihydropyrimidines.

Entry	Solvent	Catalyst (g)	Time (min)	Temperature (^o^C)	Yield^a^ (%)
1	EtOH	–	18	80	–
2	EtOH	0.01	18	80	42
3	EtOH	0.02	18	80	58
4	EtOH	0.02	30	80	78
5	EtOH	0.03	18	80	85
6	EtOH	0.04	18	80	85
7	EtOH	0.03	30	50	62
8	EtOH	0.03	18	60	69
9	EtOH	0.03	18	70	74
10	EtOH	0.03	18	Reflux	85
11	EtOH	–	15	U.S (50 W) (r.t.)	53
12	EtOH	0.01	12	U.S (50 W) (r.t.)	68
13	EtOH	0.02	10	U.S (50 W) (r.t.)	85
14	EtOH	0.02	12	U.S (50 W) (r.t.)	>95
15	EtOH	0.03	18	US (50 W) (r.t.)	85
16	H_2_O	0.02	12	US (50 W) (r.t.)	68
17	THF	0.02	12	US (50 W) (r.t.)	55
18	CH_3_CN	0.02	12	US (50 W) (r.t.)	48
19	DMSO	0.02	12	US (50 W) (r.t.)	40

^a^Isolated yield.

Based on the above optimized results, the scope and versatility of this procedure were studied by utilizing different aryl aldehyde derivatives to demonstrate the general applicability of the reaction conditions (Table 2) and the 3,4-dihydropyrimidines were obtained in good yields for aromatic aldehydes bearing electron-donating groups and excellent yields for aromatic aldehydes with lectron-withdrawing groups.

**Table 2:** Synthesis of 3,4-dihydropyrimidines.


Entry	R	Product	Yield (%)^a^	M.P. /M.P. (^o^C)^b^
1	4-H		>93	203-205/202-204^39^
2	4-Cl		>95	208-210/207-210^39^
3	4-OMe		≈90	198–200/203-205^39^
4	4-isopropyl		≈90	194-196/193-195^40^
5	4-NO		>95	203-205/206^41^
6	4-Me		≈90	206-208/208^41^
7	4-N(Me)_2_		≈90	250-252/250-253^39^
8	3-OH		90–92	165-167/165-167^39^
9	3-NO_2_		>90	223-225/225-227^39^
10	2-Cl		≈90	217-218/218^41^
11	2-OH		≈90	200-202/200-202^39^

^a^Isolated yields.

^b^Literature references.

After the success of the Biginelli reaction in producing 3,4-dihydropyrimidines in the presence of the SBA-15@Schiff‐base@Fe(III) catalyst, we used dimedone in place of ethyl acetoacetate to produce octahydroquinazolinone derivatives. The standard reaction was examined under a variety of conditions. As shown in Table 3, a maximum product yield of 75%.was obtained by 0.03 g of catalyst under reflux in EtOH (Entry 10). The excellent performance of this catalytic system was proved in the sonochemical reaction. As expected, the reaction conditions were improved by the sonication process such as higher yield (> 95%), reduced reaction time (15 min), and milder conditions at room temperature (Entry 14). The implosion of cavitation bubbles on the surface of the catalyst provides locally enough energy to improve of the reaction rate^30^. In optimizing the standard conditions for the reaction solvent, ethanol was the best solvent under ultrasound conditions. After optimizing the reaction conditions, a wide range of substituted aldehydes were applied to synthesize the octahydroquinazolinones. From Table 4, it is clear that the product yields were good to excellent with short reaction times.

**Table 3:** Optimized conditions to synthesize of octahydroquinazolinones.

Entry	Solvent	Catalyst (g)	Time (min)	Temperature (^o^C)	Yield^a^ (%)
1	EtOH	–	30	80	–
2	EtOH	0.01	30	80	38
3	EtOH	0.01	45	80	40
4	EtOH	0.02	30	80	52
5	EtOH	0.03	30	80	75
6	EtOH	0.04	30	80	75
7	EtOH	0.03	45	50	54
8	EtOH	0.03	30	60	58
9	EtOH	0.03	30	70	63
10	EtOH	0.03	30	Reflux	75
11	EtOH	–	15	U.S (50 W) (r.t.)	51
12	EtOH	0.01	15	U.S (50 W) (r.t.)	66
13	EtOH	0.02	15	U.S (50 W) (r.t.)	82
14	EtOH	0.03	15	U.S (50 W) (r.t.)	>95
15	EtOH	0.03	30	US (50 W) (r.t.)	85
16	H_2_O	0.03	15	U.S (50 W) (r.t.)	88
17	THF	0.03	15	U.S (50 W) (r.t.)	56
18	CH_3_CN	0.03	15	U.S (50 W) (r.t.)	60
19	DMSO	0.03	15	U.S (50 W) (r.t.)	80

^a^Isolated yield.

**Table 4:** Synthesis of octahydroquinazolinones.


Entry	R	Product	Yield (%)^a^	M.P. /M.P. (^o^C)^b^
1	4-H		>95	284-286/286-288^42^
2	4-Cl		>95	294-297/296-297^42^
3	4-OMe		≈90	198-199/198-202^43^
4	4-isopropyl		≈90	299-300/299-301^44^
5	4-NO		>95	222-224/224-226^43^
6	4-Me		≈90	282-284/298-300^44^
7	4-OH		≈90	273-275/274-276^44^
8	3-NO_2_		95–97	297-299/303-305^44^
9	2,4-Cl_2_		≈90	260-262/270-272^44^

^a^Isolated yields.

^b^Literature references.

**Scheme 4:**
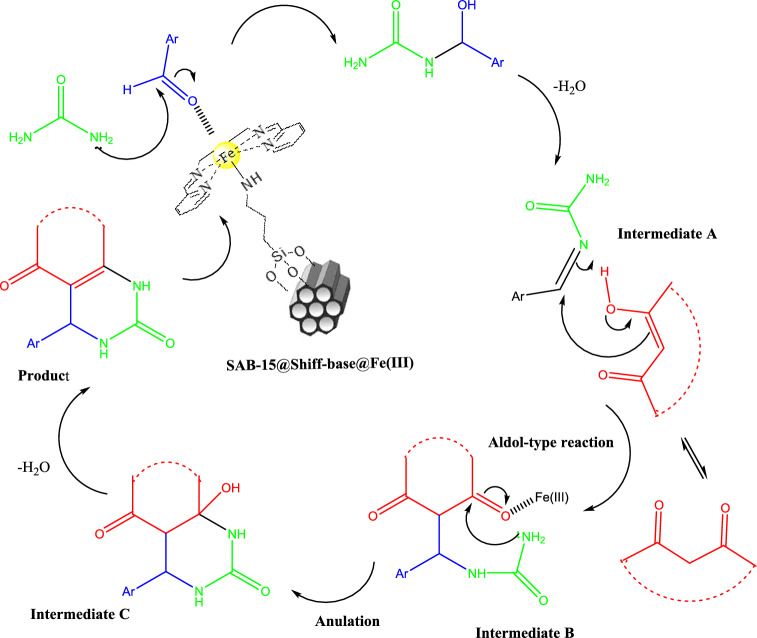
The probable mechanism for synthesis of Biginelli heterocycles.

The probable mechanism of this three component reaction is proposed in Scheme 4. According to the mechanism suggested by Neto et al.^45^. The Fe(III) immobilized SBA-15 participates as a lewis acidic catalyst in the reaction which activate the aldehyde followed by nucleophilic attack of urea molecule forming the intermediate **A**. Then, the *β*-dicarbonyl enolate adds to the imine bond of intermediate **A** to produce an open-chain intermediate ureide **B.** This intermediate is activated by the coordination of the lone pair of the oxygen atom of the carbonyl groups with the empty orbitals of Fe^3+^. Subsequently the N-nucleophilic NH_2_ attack to the carbonyl unit which in turn affords intermediate C through a cyclization reaction. Then, the cyclodehydration of intermediate to form the desired dihydropyrimidines and octahydroquinazolinones have formed the cyclodehydration of intermediate **C**.

To compare the catalytic performance of SBA-15@Schiff‐base@Fe(III) nanohybrids with the other catalysts reported in the literature, we have compared the results of these catalysts in the model reaction for the preparation of **4a** (Entries 1–10) and **6a** (Entries 11–20) under the optimization conditions (Table 5). A comparison of the results shows a better catalytic activity of SBA-15@Schiff‐base@Fe(III) to synthesize 3,4-dihydropyrimidines and octahydroquinazolinones because of their short reaction times, simple conditions, and environmental friendliness.

**Table 5:** Comparison of SBA-15@Schiff‐base@Fe(III) catalyst with other catalysts in the literature to synthesize **4a** (Entries 1–10) and **6a** (Entries 11–20).

Entry	Catalyst	Condition	Time	Yield (%)^ref^
1	PANI⋅FeCl_3_	CH_3_CN, reflux	24 h	83^41^
2	Cellulose sulfuric acid	Water, 100 °C	3.5–6.5 h	85^46^
3	Fe(III)/Al-MCM-41	CH_3_CN, reflux	4 h	85^47^
4	Cu-Schiff base-MCM-41	MeOH, RT	15 min	92^8^
5	MNP–IL–HSO_4_	solvent-free, 100 °C	30 min	95^48^
6	Bentonite/PS-SO_3_H	solvent-free, 120 °C	30 min	89^13^
7	Nano-Fe_3_O_4_-bpy-Ni(II)	MW, Solvent- free,130 °C	1 h	90^12^
8	SBA‑15@APTES	EtOH, U.S (r.t.)	1 h	Trace (This work)
9	Schiff‐base	EtOH, U.S (r.t.)	1 h	Trace (This work)
10	SBA-15@Schiff‐base@Fe(III)	EtOH, U.S (r.t.)	12 min	>93 (This work)
11	MNPs-BSAT	100 °C, solvent-free	1 h	94^11^
12	Cu/SiO_2_	EtOH, reflux	35 min	96^49^
13	VNPRP	MeOH:H_2_O (1:1)	1.5 h	90^50^
14	Nafion-H	EtOH, reflux	10 h	70^51^
15	SiO_2_-NaHSO_4_	H_2_O, 60–80 °C	1.5 h	95^52^
16	TMSCl	CH_3_CN/DMF, reflux	1.5 h	95^53^
17	VCl_3_	CH_3_CN, reflux	2 h	80^54^
18	SBA‑15@APTES	EtOH, U.S (r.t.)	1 h	Trace (This work)
19	Schiff‐base	EtOH, U.S (r.t.)	1 h	Trace (This work)
20	SBA-15@Schiff‐base@Fe(III)	EtOH, U.S (r.t.)	15 min	>95 (This work)

**Figure 9:**
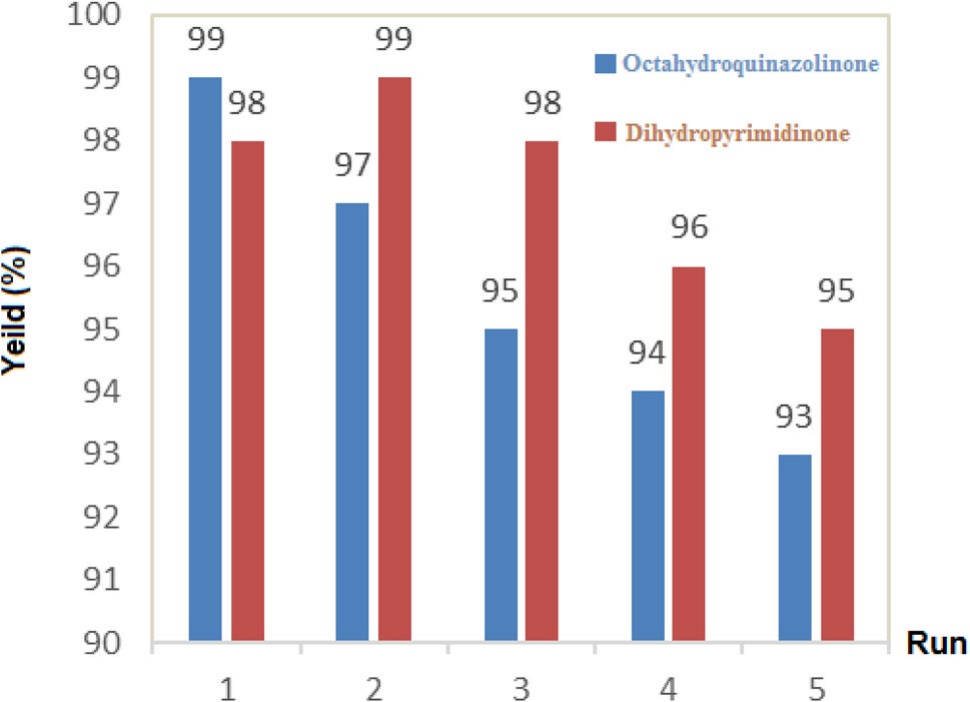
Reusability of SBA-15@Schiff‐base@Fe(III) for the synthesis of the synthesis of **(4a)** and **(6a)**.

**Figure 10:**
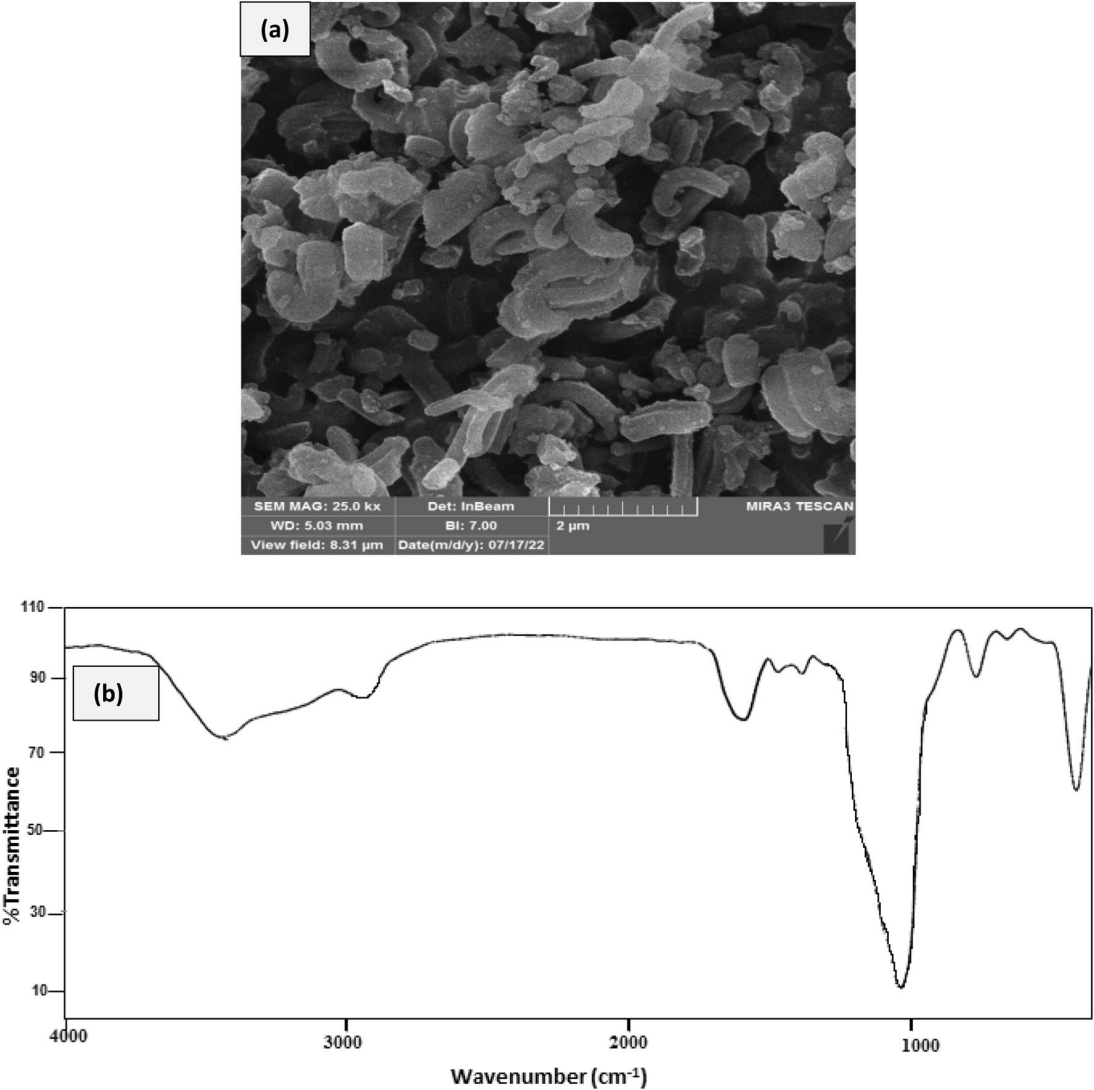
The FE-SEM (**a**) and FT-IR (**b**) of the recovred catalyst.

Figure 9 shows the high activity of the catalyst after five runs to produce Biginelli heterocycles including dihydropyrimidines and octahydroquinazolinones. Upon the completion of the reaction, the supported catalyst was filtered from the reaction medium and washed with acetone to eliminate the reaction byproducts. After five successive runs, the retrievable catalyst could be recyclable without any significant loss of activity with regard to yield product and reaction time. Furthermore, after 5 cycles of recovery, the catalyst structure had no many changes, as evident from a comparison of its FE-SEM image and FT-IR spectrum with the fresh catalyst which indicates the high stability of the catalyst (Fig. 10).

### Substances and methods

All materials were purchased with high purity from reputable commercial suppliers. ^1^H NMR spectra were recorded in DMSO-*d*_*6*_ solution by a Bruker Avance-400 MHz spectrometer, utilizing SiMe_4_ (TMS) as an internal reference at room temperature. The FT-IR spectra of the organic compounds and silica materials were recorded using an FT-IR Magna spectrometer 550 Nicolet (with KBr pellets and frequencies reported in cm^–1^). Melting points of the purified products were determined by the precise Electrothermal 9200 apparatus using open capillaries. The physical surface morphology and chemical composition of the silica frameworks were identified by a field emission scanning electron microscope (FE-SEM, MIRA3 model, TESCAN Company. and Philips XL-30FESEM) equipped with an energy-dispersive X-rey (EDX) spectrometer. The structure of SBA-15@Schiff‐base@Fe(III) was characterized using Transmission electron microscopy (TEM, Philips CM30 model) images. X-ray diffraction (XRD) test method was carried out using a Philips diffractometer Cu Kα radiation, λ = 0. 15,406 nm) from X'pert Company. The change of physical and chemical properties of the catalyst was investigated with Thermogravimetric – Differential thermal analysis (TGA-DTA) measurements using STA 503 BAEHR instrument facilitated precise measurements under a N_2_ atmosphere, employing a heating rate of 10 °C min^–1^. The completion of reactions and the purity of Biginelli compounds were tested by the thin-layer chromatography (TLC) with silica gel SILG/UV 254 plates. Sonication was performed in reactions using a multi-wave ultrasonic generator (Sonicator 3200; Bandelin, MS 73, Germany), equipped by a converter/transducer and titanium oscillator (horn), 12.5 mm diameter, processing frequency 20 kHz (effective due to uniform ultrasonic waves*)* with a maximum power output of 200 W.

### Preparation of SBA‑15@APTES

SBA-15 was synthesized as previously reported in the literature^21^. Briefly, 4 g of Pluronic P123 was dissolved in 120 mL of 2 M hydrochloric acid. The solution was stirred at room temperature for 4 h. Then, 8.5 g of tetraethoxysilane (TEOS) was added to that solution and stirred at 35 °C for 8 h. The mixture was heated at 100 °C in a autoclave. The solid product was recovered (by filtration), washed (with deionized water), and submitted to a calcination process at 550 °C for 8h. After that, 2 g of calcined SBA-15 was suspended in dry toluene (35 mL) and 2 mL of APTES was added to the suspension in an N_2_ atmosphere for 7 min. The mixture solution was refluxed at 115 °C for 48 h. The obtained solid was separated by filtration and the filtrate was washed several times with dry toluene and dried at 120 °C for 24 h in a vacuum oven. The white powder obtained was designated as SBA-15@APTES.

### Preparation of Shiff-base

In a 50 mL round-bottomed flask, a mixture of ethylenediamine (3 mmol) and pyridine-2-carbaldehyde (6 mmol) was refluxed in EtOH (6 mL) for 2 h used to synthesis of N,Nʹ-bis -(pyridin-2-ylmethylene)-ethane-1,2-diamine^23,35^. After cooling the mixture solution, the resulting residue was filtered. Then, the crude products were recrystallized in petroleum ether/ethyl acetate.

### Preparation of Shiff-base@Fe(III)

Schiff-base@Fe(III) complex was synthesized by adding Schiff-base (0.03 g) to the ethanolic solution (5 mL) of FeCl_3_.6H_2_O (0.03 g) under reflux condition for 4 h. After the end of the process, EtOH was evaporated and the precipitate was filtered off and washed with EtOH several times to obtain the Schiff-base@Fe (III) complex.

### Anchoring of shiff-base@Fe(III) complex onto SBA‑15@APTES

In a 50 mL round bottom flask, a mixture of SBA‑15@APTES (1.0 g) and Shiff-base@Fe(III) complex (0.15 g) was stirred in ethanol (10.0 mL) using a mechanical stirrer at room temperature for 24 h. The solid product was separated by centrifuging and washed three times with EtOH. Finally, the SBA-15@Schiff‐base@Fe(III) was dried at 60°C in a vacuum oven.

### The typical procedure for the synthesis of dihydropyrimidinones and octahydroquinazolinones

A mixture of aryle aldehyde (1.0 mmol), ethyl acetoacetate or dimedone (1.0 mmol), urea (1.5 mmol), and SBA-15@Schiff‐base@Fe(III) (0.03 g) was sonicated in EtOH at room temeprature. After completion of the reaction (indicated by TLC), the resulting mixture was cooled to obtain a precipitate. After filtering the crude product, it was dissolved in hot EtOH the nanocatalyst was separated by centrifugation. The solvent was evaporated to give the crude products. Then, the crude products were recrystallized in EtOH.

### Spectroscopic data for selected Biginelli heterocycles

#### 5-ethoxycarbonyl-4-phenyl-6-methyl-3,4-dihydropyrimidin-2(1*H*)-one (4a)

White solid; IR (KBr) ν (cm^−1^): 3246 (N–H), 3122 (N–H), 3056 (C–H), 2942 (C–H), 1718 (C=O), 1649 (C=C), 1468 (C=C), 1229 (C–N), 1106 (C–O). ^1^H NMR (400 MHz, DMSO-*d*_*6*_) δ 1.10 (t, 3H, *J* = 8 Hz), 2.25 (s, 3H), 3.98 (q, 2H, *J* = 8 Hz, *J* = 8 Hz), 5.14 (s, 1H), 7.23–7.35(m, 5H, *J* = 8 Hz), 7.74 (s, NH), 9.20 (s, NH) ppm.

#### 5-ethoxycarbonyl-4-(4-Cl-phenyl)-6-methyl-3,4-dihydropyrimidin-2(1*H*)-one (4b)

White solid; IR (KBr) ν (cm^−1^): 3241 (N–H), 3116 (N–H), 2929 (C–H), 1709 (C=O), 1648 (C=C), 1463 (C=C), 1223 (C–O), 1014 (C–N), 784 (C–Cl). ^1^H NMR (400 MHz, DMSO-*d*_*6*_) δ 1.10 (t, 3H, *J* = 8 Hz), 2.25 (s, 3H), 3.98 (q, 2H, *J* = 8 Hz), 5.14 (s, 1H), 7.25 (d, 2H*, J* = 8 Hz), 7.40 (d, 2H*, J* = 8 Hz) 7.78 (s, NH), 9.25 (s, NH) ppm.

#### 5-ethoxycarbonyl-4-(4-OMe-phenyl)-6-methyl-3,4-dihydropyrimidin-2(1*H*)-one (4c)

White solid; IR (KBr) ν (cm^−1^): 3241 (N–H), 3111 (N–H), 2938 (C–H), 1708 (C=O), 1648 (C=C), 1510 (C=C), 1225 (C–O), 1091 (C–N). ^1^H NMR (400 MHz, DMSO-*d*_*6*_) δ 1.20 (t, 3H,* J* = 8 Hz), 2.37 (s, 3 H), 3.81 (s, 3H), 4.10 (q, 2H, *J* = 8 Hz), 5.38 (s, 1H), 5.47 (s, NH), 6.86 (d, 2H, *J* = 8 Hz), 7.26 (d, 2H, *J* = 8 Hz), 7.48 (s, NH) ppm.

#### 5-ethoxycarbonyl-4-(4-isopropylephenyl)-6-methyl-3,4-dihydropyrimidin-2(1*H*)-one (4d)

White solid; IR (KBr) ν (cm^−1^): 3241 (N–H), 3111 (N–H), 2938 (C–H), 1708 (C=O), 1648 (C=C), 1458 (C=C), 1225 (C–N), 1091 (C–O). ^1^H NMR (400 MHz, DMSO-*d*_*6*_) δ 1.27–1.32 (m, 9H), 2.28 (s, 3H), 2.84 (septet, 1H,* J* = 8 Hz), 0.4.17 (q, 2H, *J* = 4 Hz), 5.66 (s, 1H), 6.29 (s, NH), 7.20 (d, 2H, *J* = 8 Hz), 7.30 (d, 2H, *J* = 8 Hz) 8.54 (s, NH) ppm.

#### 5-ethoxycarbonyl-4-(4-NO_2_-phenyl)-6-methyl-3,4-dihydropyrimidin-2(1*H*)-one (4e)

White solid; IR (KBr) ν (cm^−1^): 3347 (N–H), 3226 (N–H), 3111 (C–H aromatic) 2973 (C–H aliphatic), 1698 (C=O) 1637 (C=C), 1456 (N–O, symmetric), 1535 (N–O, asymmetric), 1341 (C–N), 1098 (C–O). ^1^H NMR (400 MHz, DMSO-*d*_*6*_) δ 1.10 (t, 3 H, *J* = 8 Hz), 2.27 (s, 3 H), 3.98 (q, 2 H, *J* = 8 Hz), 5.27 (s, 1H), 7.51 (d, 2 H, *J* = 8 Hz), 7.90 (s, NH), 8.22 (d, 2 H, *J* = 12 Hz), 9.36 (s, NH) ppm.

#### 5-ethoxycarbonyl-4-(4-Me-phenyl)-6-methyl-3,4-dihydropyrimidin-2(1*H*)-one (4f)

White solid; IR (KBr) ν (cm^−1^): 3433 (N–H), 3242 (N–H), 3116 (C–H aromatic), 2975 (C–H aliphatic), 1709 (C=O), 1647 (C=C), 1221 (C–N), 1089 (C–O). ^1^H NMR (400 MHz, DMSO-*d*_*6*_) δ 1.11 (t, 3H, *J* = 8 Hz), 2.24 (s, 3 H), 2.45 (s, 3H), 3.98 (q, 2H, *J* = 8 Hz), 5.10 (s, 1H), 7.17 (d, 2H, *J* = 8 Hz),7.22 (d, 2 H, *J* = 8 Hz), 7.73 (s, NH), 9.21 (s, NH) ppm.

#### 5-ethoxycarbonyl-4-(4-dimethylamino)phenyl)-6-methyl-3,4-dihydropyrimidin-2(1*H*)-one (4g)

White solid; IR (KBr) ν (cm^−1^): 3357 (N–H), 3216 (N–H), 3102 (C–H Aromatic) 2962 (C–H aliphatic), 1697 (C=O), 1623 (C=C), 1276 (C–N), 1156 (C–O). ^1^H NMR (400 MHz, DMSO-*d*_6_) δ 1.12 (t, 3H, *J* = 8 Hz), 2.23 (s, 3H), 2,85 (s, 6H), 3.98 (q, 2H, *J* = 8 Hz), 5.03(s, 1 H), 6.66 (d, 2H, *J* = 8 Hz), 7.04 (d, 2H, *J* = 8 Hz), 7.59 (s, NH), 9.09 (s, NH) ppm.

#### 5-ethoxycarbonyl-4-(3-OH-phenyl)-6-methyl-3,4-dihydropyrimidin-2(1*H*)-one (4h)

White solid; IR (KBr) ν (cm^−1^): 3517 (O–H), 3243 (N–H), 3199 (N–H), 2978 (C–H), 1723 (C=O), 1639 (C=C), 1222(C–N), 1092 (C–O). ^1^H-NMR (DMSO-*d*_6_, 400 MHz) d (ppm): 1.10 (t, 3H*, J* = 8 Hz, 3H,), 2.22 (s, 3H), 3.98 (q, 2H, *J* = 8), 5.08 (s, 1H), 6.58–6.70 (m, 3H), 7.08 (dd, *J* = 15 Hz, 1H), 7.68 (s, 1H, NH), 9.15 (s, 1H, NH), 9.36 (1H, OH).

#### 5-ethoxycarbonyl-4-(3-NO_2_-phenyl)-6-methyl-3,4-dihydropyrimidin-2(1*H*)-one (4i)

White solid; IR (KBr) ν (cm^−1^): 3217 (NH), 3101 (NH), 2964 (C–H aliphatic), 1707 (C=O), 1628 (C=C), 1374 (C–N), 1088 (C–O). ^1^H NMR (400 MHz, DMSO-*d*_6_) δ 1.08 (t, *J* = 8 Hz, 3H), 2.25 (s, 3H), 3.99 (q, 2H, *J* = 8), 5.28 (s, 1H), 7.63 (d, *J* = 7.6 Hz, 1H), 7.68 (dd, *J* = 15 Hz, 1H), 7.91 (s, 1H, NH), 8.07 (s, 1H), 8.13 (d, *J* = 7.6 Hz, 1H), 9.38 (s, 1H, NH).

#### 5-ethoxycarbonyl-4-(2-Cl-phenyl)-6-methyl-3,4-dihydropyrimidin-2(1*H*)-one (4j)

White solid; IR (KBr) ν (cm^−1^): 3241 (N–H), 3106 (N–H), 2973 (C–H), 1700 (C=O), 1647 (C=C), 1550 (C=C), 1224 (C–N), 1083 (C–O). ^1^H NMR (400 MHz, DMSO-*d*_*6*_) δ 1.00 (t, 3H, *J* = 8 Hz), 2.31 (s, 3H), 3.90 (q, 2H, *J* = 8 Hz), 5.63 (s, 1H), 7.25–7.42 (m, 4H), 7.71 (s, NH), 9.28 (s, NH) ppm.

#### 5-ethoxycarbonyl-4-(2-OH-phenyl)-6-methyl-3,4-dihydropyrimidin-2(1*H*)-one (4k)

White solid; IR (KBr) ν (cm^−1^): 3410 (OH, wide), 3278 (N–H), 1685 (C=O), 1612 (C=C), 1462 (C=C), 1235 (C–N), 1090 (C–O). ^1^H NMR (400 MHz, DMSO-*d*_*6*_) δ 1.30 (t, 3H, *J* = 8 Hz), 2.28 (s, 3H), 4.17 (q, 2H, *J* = 8 Hz), 5.78 (s, 1H), 6.70 (s, NH), 6.88 (d.1H, *J* = 8Hz), 7.01 (s, OH), 7.07(t, 1H, *J* = 8 Hz), 7.15 (t, 1H, *J* = 8 Hz), 7.41(d, 1H, *J* = 8 Hz), 6.70 (s, NH), 8.54 (s, NH) ppm.

#### 4-phenyl-7,7-dimethyl-4,6,7,8-tetrahydro-1*H*,3*H*-quinazoline-2,5-dione (6a)

White solid; IR (KBr) ν (cm^−1^): 3318 (N–H), 3220 (N–H), 3105 (C–H), 2961 (C–H), 1702 (C=O), 1626 (C=C), 1508 (C=C), 1236 (C–N). ^1^H NMR (400 MHz, DMSO-*d*_*6*_) δ 0.89, (s, 3H), 1.02 (s, 3H), 2.03 (d, 1H, *J* = 16 Hz), 2.20 (d, 1H, *J* = 16 Hz), 2.27 (d, 1H, *J* = 20 Hz), 2.41 (d, 1H, *J* = 20 Hz), 5.15(s, 1H), 7.23–7.33 (m, 5H), 7.77 (s, NH), 9.47 (s, NH) ppm.

#### 4-(4-Chloro-phenyl)-7,7-dimethyl-4,6,7,8-tetrahydro-1*H*,3*H*-quinazoline-2,5-dione (6b)

White solid; IR (KBr) ν (cm^−1^): 3423 (N–H), 3220 (N–H), 3106 (C–H), 2962 (C–H), 1627 (C=O), 1508 (C=C), 1235 (C–N). ^1^H NMR (400 MHz, DMSO-*d*_*6*_) δ 0.88 (s, 3H), 1.02 (s, 3H), 2.07 (d, *J* = 20 Hz, 1H), 2.24 (d, *J* = 20 Hz, 1H), 2.40 (d, J = 12 Hz, 2H), 5.19 (s, 1H), 7.21–7.24 (m, 2H), 7.41–7.43 (m, 2H), 9.68 (s, NH), 10.60 (s, NH) ppm.

#### 4-(4-Methoxy-phenyl)-7,7-dimethyl-4,6,7,8-tetrahydro-1*H*,3*H*-quinazoline-2,5-dione (6c)

White solid; IR (KBr) ν (cm^−1^): 3318 (N–H), 3247 (N–H), 2954(C–H), 1674 (C=O), 1608 (C=C), 1237 (C–N). ^1^H NMR (400 MHz, DMSO-*d*_*6*_) δ 0.89 (s, 3H), 1.00 (s, 3H), 2.00 (d, *J* = 16 Hz, 1H), 2.18 (d, *J* = 16 Hz, 1H), 2.24 (d, *J* = 20 Hz, 1H), 2.39 (d, *J* = 20 Hz, 1H), 3.70 (s, 3H), 5.07 (s, 1H), 6.85 (d, *J* = 8 Hz, 2H), 7.13 (d, *J* = 8 Hz, 2H), 7.70 (s, NH), 9.43 (s, NH) ppm.

#### 4-(4-isopropylephenyl)-7,7-dimethyl-4,6,7,8-tetrahydro-1*H*,3*H*-quinazoline-2,5-dione (6d)

White solid; IR (KBr) ν (cm^−1^): 3357 (N–H), 3216 (N–H), 3102 (C–H aromatic), 2962 (C–H aliphatic), 1697 (C=O), 1623 (C=C), 1525 (C=C), 1236 (C–N). ^1^H NMR (400 MHz, DMSO-*d*_*6*_) δ 0.92 (s, 3H), 1.02 (s, 3H), 1.17 (d, 6H, *J* = 8 Hz), 2.03 (d, 1H, *J* = 16 Hz), 2.19 (d, 1H, *J* = 16 Hz), 2.28 (d, 1H, *J* = 20 Hz), 2.41 (d, 1H, *J* = 16 Hz), 2.84 (septet, 1H, *J* = 8 Hz) 5.11(s, 1H), 7.14 (d, 2H, *J* = 8 Hz), 7.18 (d, 2H, *J* = 8 Hz), 7.70 (s, NH), 9.43 (s, NH) ppm.

#### 4-(4-Nitro-phenyl)-7,7-dimethyl-4,6,7,8-tetrahydro-1*H*,3*H*-quinazoline-2,5-dione (6e)

White solid; IR (KBr) ν (cm^−1^): 3327 (N–H), 3245 (N–H), 2961 (C–H), 1671 (C=O), 1525 (C=C), 1236 C–N). ^1^H NMR (400 MHz, DMSO-*d*_*6*_) δ 0.86 (s, 3H), 1.02 (s, 3H), 2.03 (d, *J* = 16 Hz, 1H), 2.21 (d, *J* = 16 Hz, 1H), 2.28 (d, *J* = 16 Hz, 1H), 2.43 (d, *J* = 16 Hz, 1H), 5.30 (s, 1H), 7.51 (d, *J* = 8 Hz, 2H), 7.93 (s, NH), 8.21 (d, *J* = 8 Hz, 2H), 9.65 (s, NH) ppm.

#### 4-(4-Methylphenyl)-7,7-dimethyl-4,6,7,8-tetrahydro-1*H*,3*H*-quinazoline-2,5-dione (6f)

White solid; IR (KBr) ν (cm^−1^): 3340 (N–H), 3254 (N–H), 3100 (C–H aromatic), 2957 (C–H aliphatic), 1698 (C=O), 1627 (C=C), 1499 (C=C), 1235 (C–N). ^1^H NMR (400 MHz, DMSO-*d*_*6*_) δ 0.89, (s, 3H), 1.02 (s, 3H), 2.01 (d, 1H, *J* = 16 Hz), 2.19 (d, 1H, *J* = 16 Hz),2.25 (s, 3H) 2.27 (d, 1H, *J* = 12 Hz), 2.40(d, 1H, *J* = 20 Hz), 5.10 (s, 1H), 7.11 (s, 4H), 7.72 (s, NH), 9.43 (s, NH) ppm.

#### 4-(4-Hydroxy-phenyl)-7,7-dimethyl-4,6,7,8-tetrahydro-1*H*,3*H*-quinazoline-2,5-dione (6g)

White solid; IR (KBr) ν (cm^−1^): 3247 (N–H), 3411 (N–H), 2960 (C–H), 1650 (C=O), 1525 (C=C), 1374 (C–N). ^1^H NMR (400 MHz, DMSO-*d*_*6*_) δ 0.89 (s, 3H), 1.00 (s, 3H), 2.00 (d, *J* = 16 Hz, 1H), 2.17 (d, *J* = 16 Hz, 1H), 2.24 (d, *J* = 16 Hz, 1H), 2.38 (d, *J* = 16 Hz, 1H), 5.02 (s, 1H), 6.66 (d, *J* = 8 Hz, 2H), 7.01 (d, *J* = 8 Hz, 2H), 7.65 (s, OH), 9.33 (s, NH), 9.39 (s, NH) ppm.

#### 4-(3-Nitro-phenyl)-7,7-dimethyl-4,6,7,8-tetrahydro-1*H*,3*H*-quinazoline-2,5-dione (6h)

White solid; IR (KBr) ν (cm^−1^): 3357 (N–H), 3216 (N–H), 3102 (C–H aromatic), 2962 (C–H aliphatic), 1697 (C=O), 1623, (C=C), 1445 (C=C), 1525 (asymmetric, N = O), 1376 (symmetric, N = O), 1236 (C–N). ^1^H NMR (400 MHz, DMSO-*d*_*6*_) δ 70.91(s, 3H), 1.04 (s, 3H), 2.05 (d, 1H, *J* = 20 Hz), 2.22 (d, 1H, *J* = 16 Hz), 2.30 (d, 1H, *J* = 20 Hz), 2.45 (d, 1H, *J* = 20 Hz), 5.33 (s, 1H), 7.63–7.72 (m, 2H), 7.93 (s, NH), 8.07–8.13 (m, 2H), 9.64 (s, NH) ppm.

#### 4-(2,4-dichlorophenyl)-7,7-dimethyl-4,6,7,8-tetrahydro-1*H*,3*H*-quinazoline-2,5-dione (6i)

White solid; IR (KBr) ν (cm^−1^): 3318 (N–H), 3220 (N–H), 3105 (C–H aromatic), 2961 (C–H aliphatic), 1702 (C=O), 1626 (C=C), 1508 (C=C), 1236 (C–N). 1H NMR (400 MHz, DMSO-*d*_*6*_) δ 0.95 (s, 3H), 1.02 (s, 3H), 1.98 (d, 1H, *J* = 16 Hz), 2.16 (d, 1H, *J* = 16Hz), 2.32 (d, 1H, *J* = 20 z), 2.43 (d, 1H, *J* = 20 Hz), 5.53(s, 1H), 7.29 (d, 1H, *J* = 8 Hz), 7.40 (d, 1H, *J* = 8 Hz) 7.54 (s, 1H), 7.75 (s, NH), 9.58 (s, NH) ppm.

## Conclusion

In the current study, we have developed a modified and validated methodology to synthesize hydroquinazoline-2,5-diones and 3,4-dihydropyrimidin-2(1*H*)-ones. using Siff-base@Fe(III) anchored SBA-15 as a catalyst via ultrasound technology with approachable yields of products. Reduced reaction times, simple set of workup, easy availability, and recyclability of catalyst are some of the obvious positive points of the present synthetic method.

### Supplementary Information

Supplementary Information.

## Data Availability

This published article and its Supplementary Information file include all data generated or analyzed during this research.
