# Supplementary material for: Synthesis of 3,4-dihydropyrimidines and octahydroquinazolinones by SBA-15 supported schiff-base iron (III) complex as durable and reusable catalyst under ultrasound irradiation
Source: Sci Rep. 2024 Jun 27;14:14810. doi: 10.1038/s41598-024-65519-x (PMC11208551; doi:10.1038/s41598-024-65519-x)
Supplement: Supplementary file 1 — Supplementary Information. [file 41598_2024_65519_MOESM1_ESM.docx]

**Synthesis of 3,4-Dihydropyrimidines and Octahydroquinazolinones by SBA-15 Supported Schiff-base Iron (III) Complex as Durable and Reusable Catalyst under ultrasound irradiation**

Zeynab Balali ^1^, Javad Safaei-Ghomi^*1^ , Elahe Mashhadi^1^

^1^Department of Organic Chemistry, Faculty of Chemistry, University of Kashan, Kashan, I. R. Iran,

*E-mail address: [safaei@kashanu.ac.ir](mailto:safaei@kashanu.ac.ir).

**N,N'-bis-(pyridin-2-ylmethylene)-ethane-1,2-diamine**

Brown solid; IR (KBr) ν (cm^−1^): 1644 (C═N). ^1^H NMR (250 MHz, CDCl_3_) δ 8.61 (ddd, *J* = 4.8, 1.7, 1.0 Hz, 1H), 8.41 (s, 1H), 7.97 (dt, *J* = 7.9, 1.1 Hz, 1H), 7.71 (td, *J* = 7.7, 1.8 Hz, 1H), 7.35 – 7.28 (m, 1H), 7.26 (s, 1H), 4.06 (s, 1H) ppm.


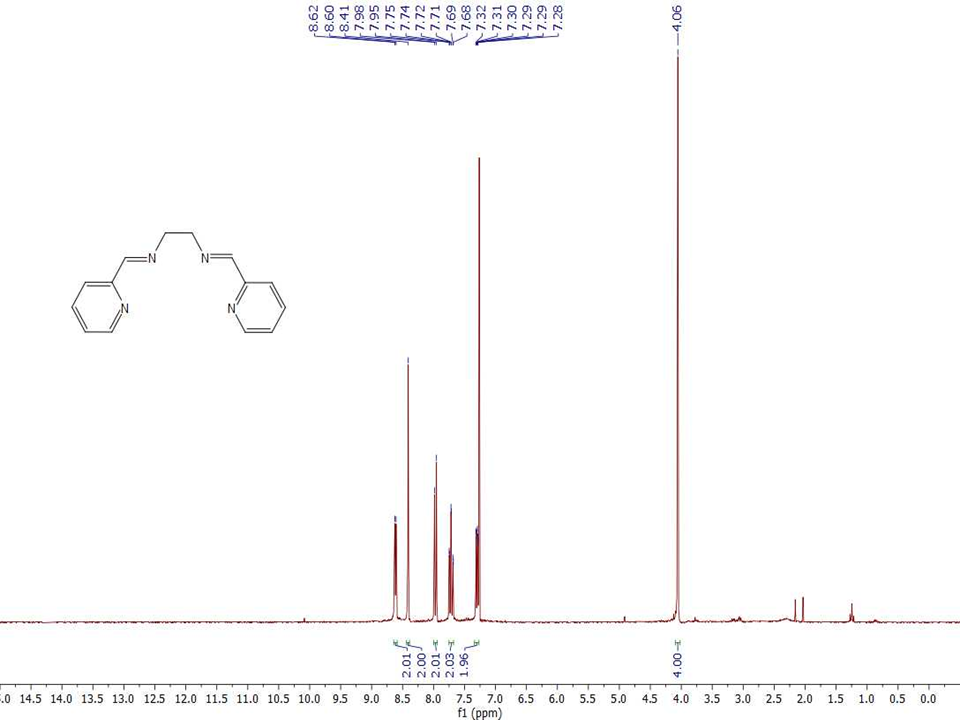


Figure S1: ^1^H NMR spectrum of Shiff-base

**5-Ethoxycarbonyl-4-phenyl-6-methyl-3,4-dihydropyrimidin-2(1*H*)-one** (4a)

White solid; IR (KBr) ν (cm^−1^): 3246 (N-H), 3122 (N-H), 3056 (C-H), 2942 (C-H), 1718 (C=O), 1649 (C=C), 1468 (C=C), 1229 (C-N), 1106 (C-O). ^1^H NMR (400 MHz, DMSO-*d_6_*) δ 1.10 (t, 3H, *J* = 8 Hz), 2.25 (s, 3H), 3.98 (q, 2H, *J* = 8 Hz, *J* = 8 Hz), 5.14 (s, 1H), 7.23-7.35(m, 5H, *J* = 8 Hz), 7.74 (s, NH), 9.20 (s, NH) ppm.


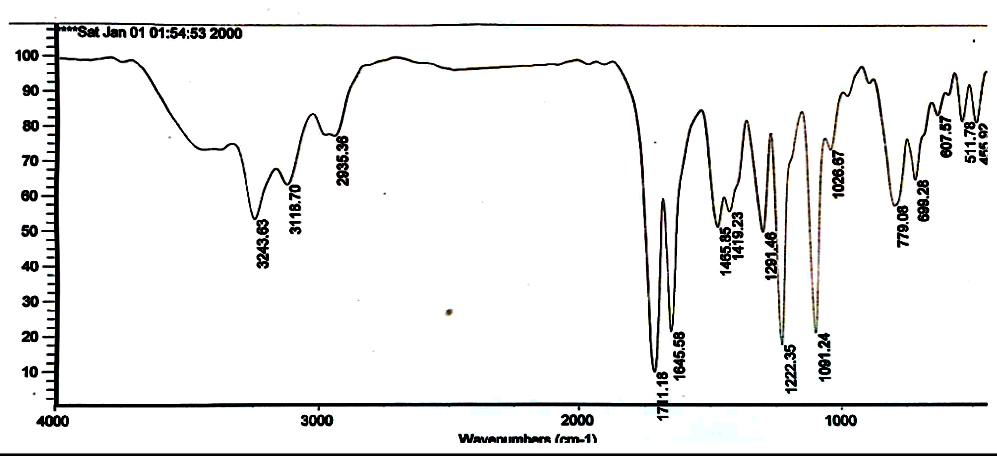


Figure S2: FT-IR spectrum of compound 4a

Figure S3: ^1^H NMR spectrum of compound 4a

**5-Ethoxycarbonyl-4-(4-Cl-phenyl)-6-methyl-3,4-dihydropyrimidin-2(1*H*)-one** (4b)

White solid; IR (KBr) ν (cm^−1^): 3241 (N-H), 3116 (N-H), 2929 (C-H), 1709 (C=O), 1648 (C=C), 1463 (C=C), 1223 (C-O), 1014 (C-N), 784 (C-Cl). ^1^H NMR (400 MHz, DMSO-*d_6_*) δ 1.10 (t, 3H, *J* = 8 Hz), 2.25 (s, 3H), 3.98 (q, 2H, *J* = 8 Hz), 5.14 (s, 1H), 7.25 (d, 2H*, J* = 8 Hz), 7.40 (d, 2H*, J* = 8 Hz) 7.78 (s, NH), 9.25 (s, NH) ppm.


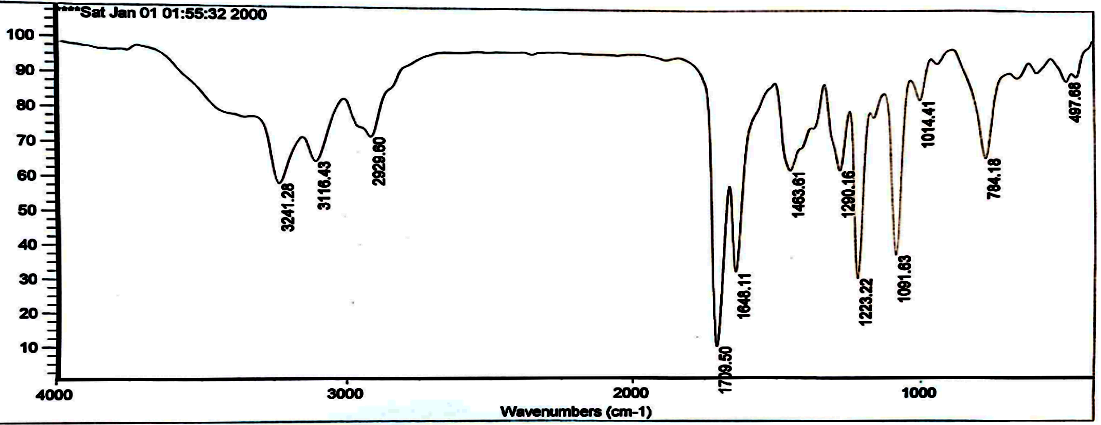


Figure S4: FT-IR spectrum of compound 4b

Figure S5: ^1^H NMR spectrum of compound 4b

**5-Ethoxycarbonyl-4-(4-OMe-phenyl)-6-methyl-3,4-dihydropyrimidin-2(1*H*)-one** (4c)

White solid; IR (KBr) ν (cm^−1^): 3241 (N-H), 3111 (N-H), 2938 (C-H), 1708 (C=O), 1648 (C=C), 1510 (C=C), 1225 (C-O), 1091 (C-N). ^1^H NMR (400 MHz, DMSO-*d_6_*) δ 1.20 (t, 3H, *J* = 8 Hz), 2.37 (s, 3 H), 3.81 (s, 3H), 4.10 (q, 2H, *J* = 8 Hz), 5.38 (s, 1H), 5.47 (s, NH), 6.86 (d, 2H, *J* = 8 Hz), 7.26 (d, 2H, *J* = 8 Hz), 7.48 (s, NH) ppm.


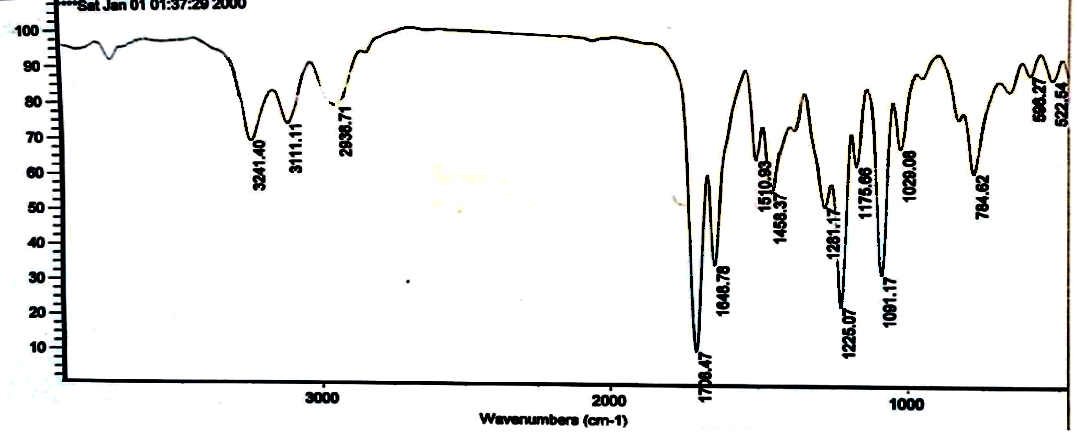


Figure S6: FT-IR spectrum of compound 4c

Figure S7: ^1^H NMR spectrum of compound 4c

**5-Ethoxycarbonyl-4-(4-isopropylephenyl)-6-methyl-3,4-dihydropyrimidin-2(1*H*)-one** (4d)

White solid; IR (KBr) ν (cm^−1^): 3241 (N-H), 3111 (N-H), 2938 (C-H), 1708 (C=O), 1648 (C=C), 1458 (C=C), 1225 (C-N), 1091 (C-O). ^1^H NMR (400 MHz, DMSO-*d_6_*) δ 1.27-1.32 (m, 9H), 2.28 (s, 3H), 2.84 (septet, 1H, *J* = 8 Hz), .4.17 (q, 2H, *J* = 4 Hz), 5.66 (s, 1H), 6.29 (s, NH), 7.20 (d, 2H, *J* = 8 Hz), 7.30 (d, 2H, *J* = 8 Hz) 8.54 (s, NH) ppm.


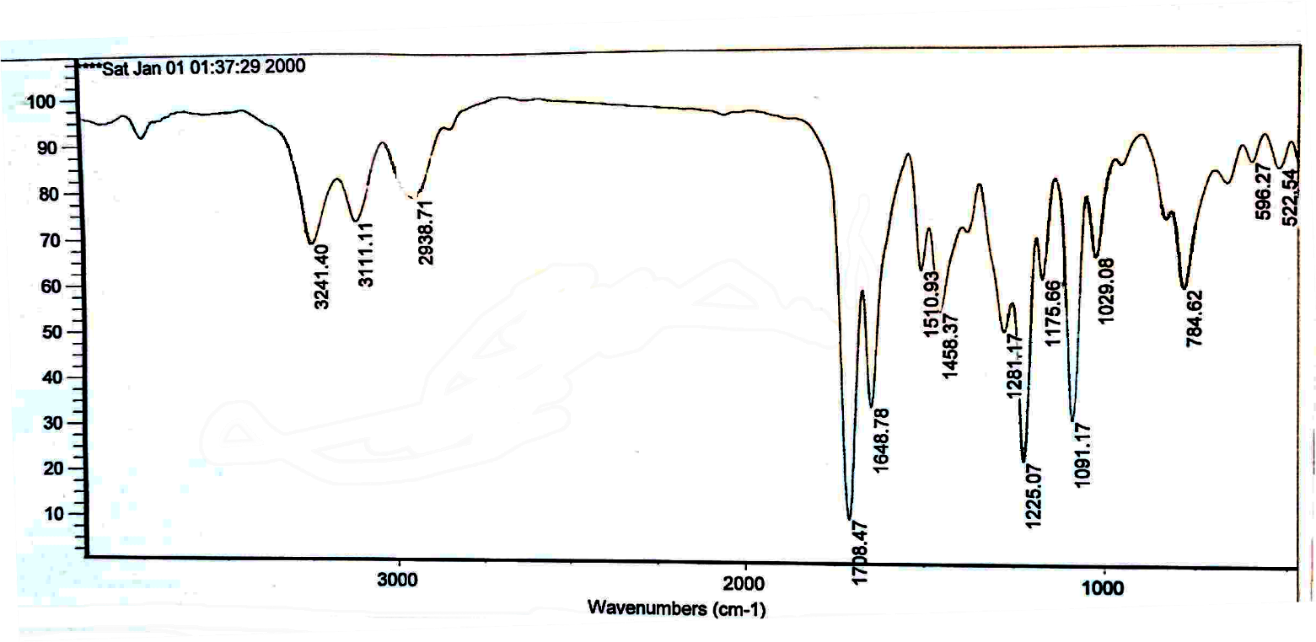


Figure S8: FT-IR spectrum of compound 4d

Figure S9: : ^1^H NMR spectrum of compound 4d

**5-Ethoxycarbonyl-4-(4-NO_2_-phenyl)-6-methyl-3,4-dihydropyrimidin-2(1*H*)-one** (4e)

White solid; IR (KBr) ν (cm^−1^): 3347 (N-H), 3226 (N-H), 3111 (C-H Aromatic) 2973 (C-H aliphatic), 1698 (C=O) 1637 (C=C), 1456 (N-O, symmetric), 1535 (N-O, asymmetric), 1341 (C-N), 1098 (C-O). ^1^H NMR (400 MHz, DMSO-*d_6_*) δ 1.10 (t, 3H, *J*=8 Hz), 2.27 (s, 3 H), 3.98 (q,2H, *J*=8 Hz), 5.27 (s, 1H), 7.51 (d,2H, *J*=8 Hz), 7.90 (s, NH), 8.22 (d, 2H, *J*=12 Hz), 9.36 (s, NH) ppm.


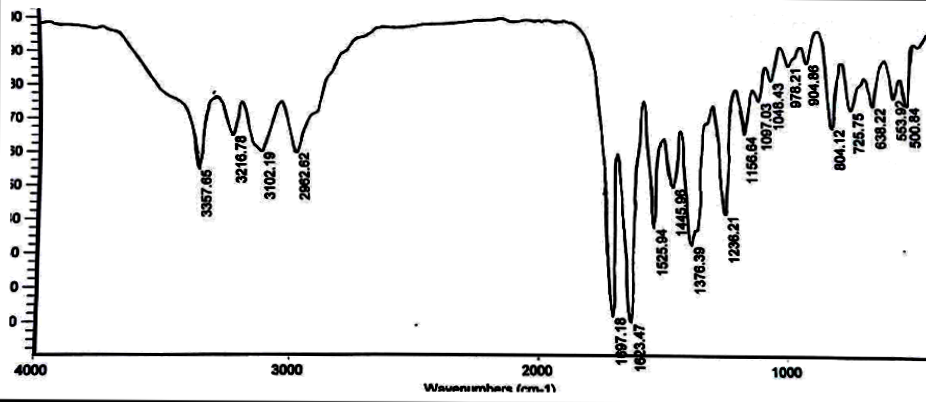


Figure S10: FT-IR spectrum of compound 4e

Figure 11: ^1^H NMR spectrum of compound 4e

**5-Ethoxycarbonyl-4-(4-Me-phenyl)-6-methyl-3,4-dihydropyrimidin-2(1*H*)-one** (4f)

White solid; IR (KBr) ν (cm^−1^): 3433 (N-H), 3242 (N-H), 3116 (C-H Aromatic), 2975 (C-H Aliphatic), 1709 (C=O), 1647 (C=C), 1221 (C-N), 1089 (C-O). ^1^H NMR (400 MHz, DMSO-*d_6_*) δ 1.11 (t, 3H, *J* = 8 Hz), 2.24 (s, 3 H), 2.45 (s, 3H), 3.98 (q, 2H, *J* = 8 Hz), 5.10 (s, 1H), 7.17 (d, 2H, *J* = 8 Hz),7.22 (d, 2 H, *J* = 8 Hz), 7.73 (s, NH), 9.21 (s, NH) ppm


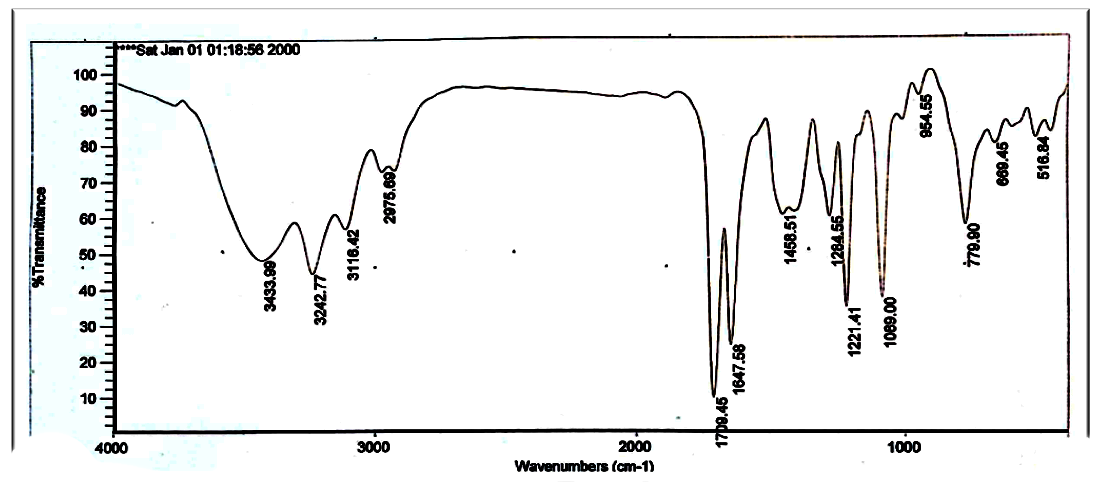


Figure S12: FT-IR spectrum of compound 4f

Figure S13: : ^1^H NMR spectrum of compound 4f

**5-Ethoxycarbonyl-4-(2-OH-phenyl)-6-methyl-3,4-dihydropyrimidin-2(1*H*)-one** (4l)

White solid; IR (KBr) ν (cm^−1^): 3410 (OH, wide), 3278 (N-H), 1685 (C=O), 1612 (C=C), 1462 (C=C), 1235 (C-N), 1090 (C-O). ^1^H NMR (400 MHz, DMSO-*d_6_*) δ 1.30 (t, 3H, *J* = 8 Hz), 2.28 (s, 3H), 4.17 (q, 2H, *J* = 8 Hz), 5.78 (s, 1H), 6.70 (s, NH), 6.88 (d.1H, *J* = 8Hz), 7.01 (s, OH), 7.07(t, 1H, *J* = 8 Hz), 7.15 (t, 1H, *J* = 8 Hz), 7.41(d, 1H, *J* = 8 Hz), 6.70 (s, NH), 8.54 (s, NH) ppm.


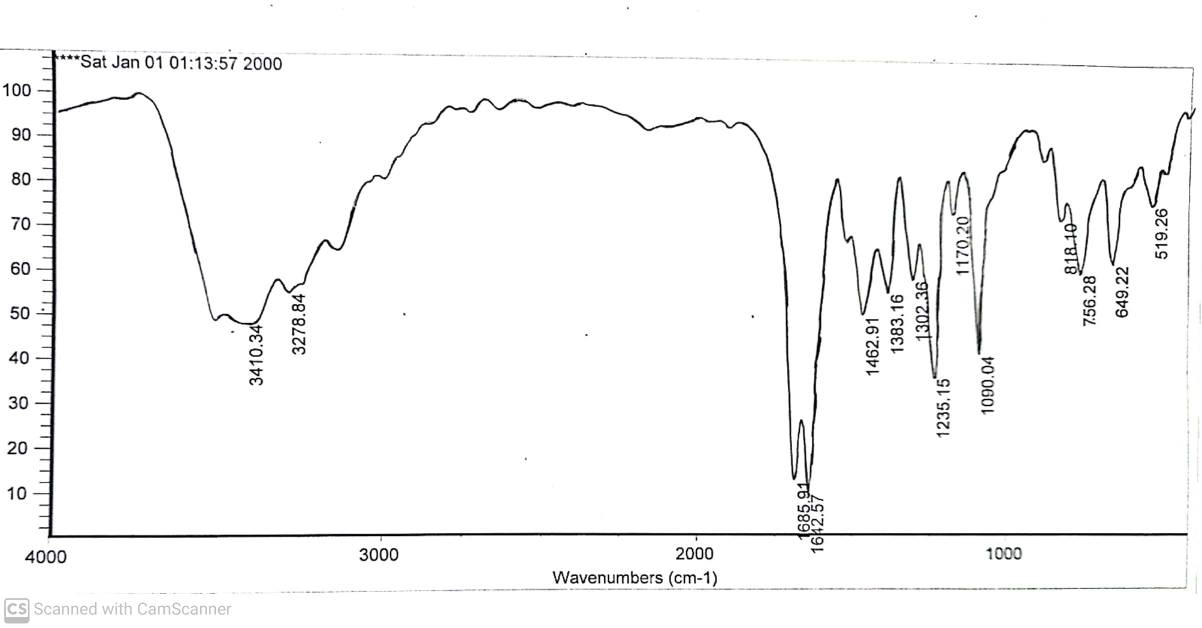


Figure S14: FT-IR spectrum of compound 4l

Figure S15: : ^1^H NMR spectrum of compound 4l

**5-Ethoxycarbonyl-4-(2-Cl-phenyl)-6-methyl-3,4-dihydropyrimidin-2(1*H*)-one** (4m)

White solid; IR (KBr) ν (cm^−1^): 3241 (N-H), 3106 (N-H), 2973 (C-H), 1700 (C=O), 1647 (C=C), 1550 (C=C), 1224 (C-N), 1083 (C-O). ^1^H NMR (400 MHz, DMSO-*d_6_*) δ 1.00 (t, 3H, *J* = 8 Hz), 2.31 (s, 3H), 3.90 (q, 2H, *J*=8Hz), 5.63 (s, 1H), 7.25-7.42 (m, 4H), 7.71 (s, NH), 9.28 (s, NH) ppm.


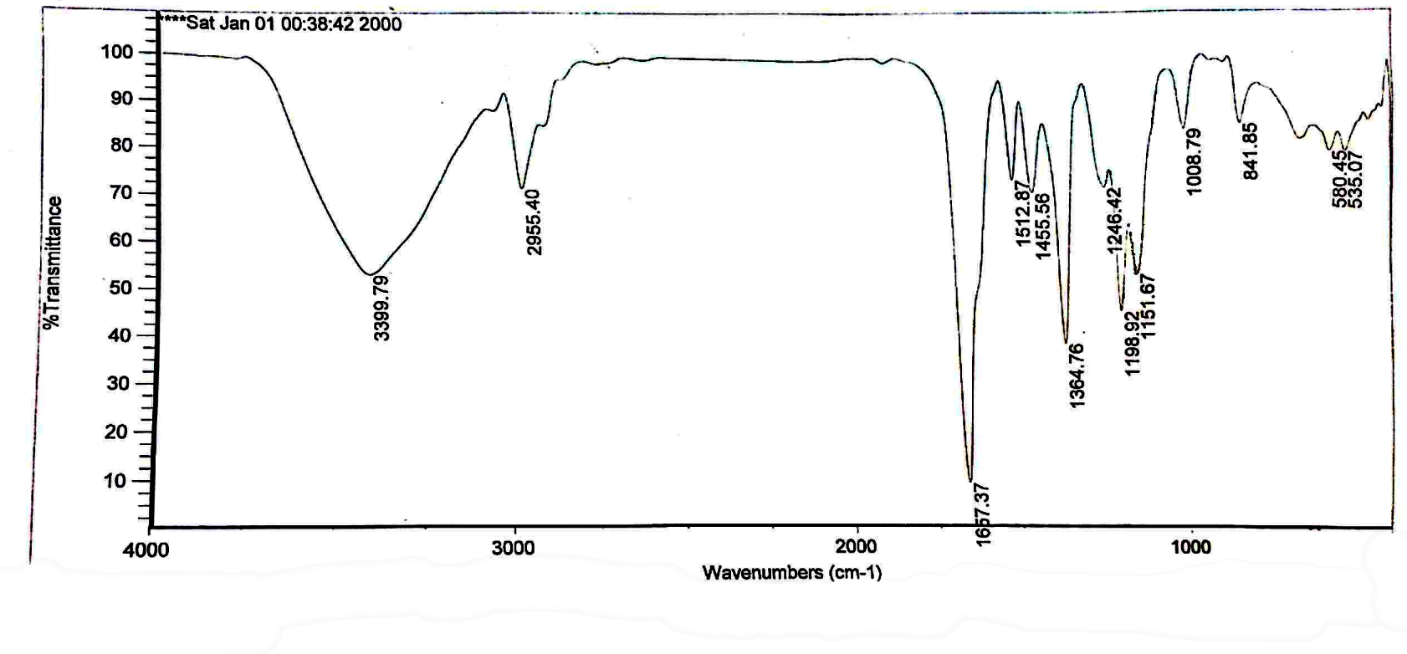


Figure S16: FT-IR spectrum of compound 4m

Figure S17: ^1^H NMR spectrum of compound 4m

**7,7-dimethyl-4-phenyl-3,4,7,8-tetrahydroquinazoline-2,5(1H,6H)-dione** (6a)

White solid; IR (KBr) ν (cm^−1^): 3318 (N-H), 3220 (N-H), 3105 (C-H), 2961 (C-H), 1702 (C=O), 1626 (C=C), 1508 (C=C), 1236 (C-N). ^1^H NMR (400 MHz, DMSO-*d_6_*) δ 0.89, (s, 3H), 1.02 (s, 3H), 2.03 (d, 1H, *J* =16 Hz), 2.20 (d, 1H, *J* =16 Hz), 2.27 (d, 1H, *J* = 20 Hz), 2.41 (d, 1H, *J* =20 Hz), 5.15(s, 1H), 7.23-7.33 (m, 5H), 7.77 (s, NH), 9.47 (s, NH) ppm.


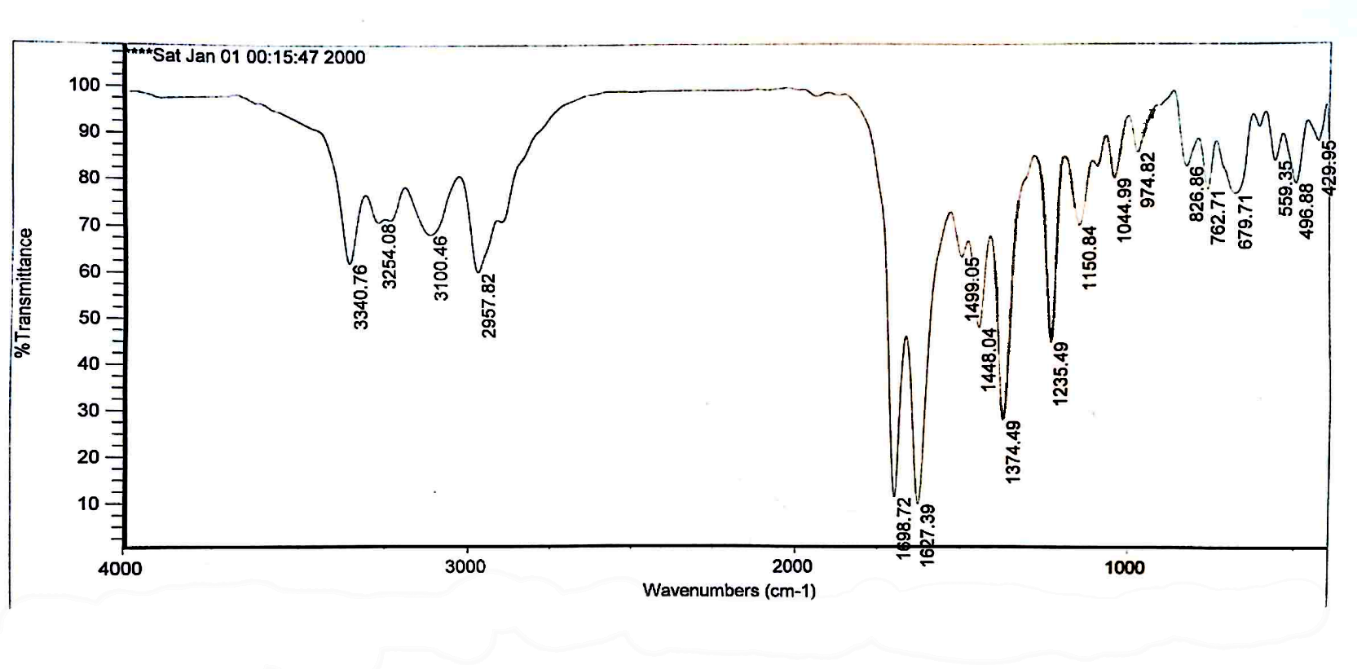


Figure S18: FT-IR spectrum of compound 6a

Figure S19: ^1^H NMR spectrum of compound 6a

**7,7-dimethyl-4-(4-isopropylephenyl)-3,4,7,8-tetrahydroquinazoline-2,5(1H,6H)-dione** (6d)

White solid; IR (KBr) ν (cm^−1^): 3357 (N-H), 3216 (N-H), 3102 (C-H Aromatic), 2962 (C-H Aliphatic), 1697 (C=O), 1623 (C=C), 1525 (C=C), 1236 (C-N). ^1^H NMR (400 MHz, DMSO-*d_6_*) δ 0.92 (s, 3H), 1.02 (s, 3H), 1.17 (d, 6H, *J* = 8 Hz), 2.03 (d, 1H, *J* =16 Hz), 2.19 (d, 1H, *J* =16 Hz), 2.28 (d, 1H, *J* =20 Hz), 2.41 (d, 1H, *J* =16 Hz), 2.84 (septet, 1H, *J* = 8 Hz) 5.11(s, 1H), 7.14 (d, 2H, *J* = 8 Hz), 7.18 (d, 2H, *J* = 8 Hz), 7.70 (s, NH), 9.43 (s, NH) ppm.


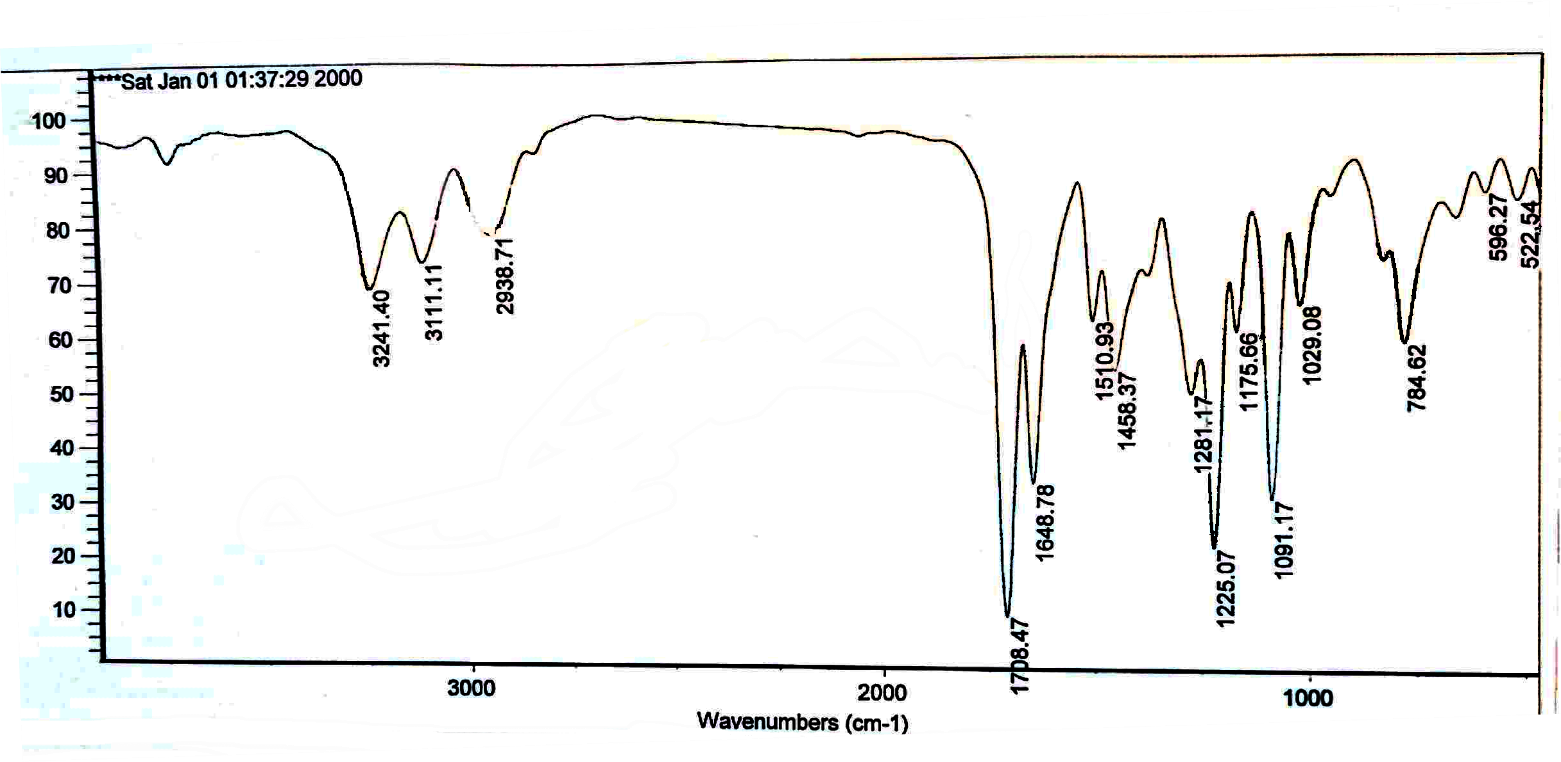


Figure S20: FT-IR spectrum of compound 6d

Figure S21: ^1^H NMR spectrum of compound 6d

**7,7-dimethyl-4-(4-Methylphenyl)-3,4,7,8-tetrahydroquinazoline-2,5(1H,6H)-dione** (6f)

White solid; IR (KBr) ν (cm^−1^): 3340 (N-H), 3254 (N-H), 3100 (C-H Aromatic), 2957 (C-H Aliphatic), 1698 (C=O), 1627 (C=C), 1499 (C=C), 1235 (C-N). ^1^H NMR (400 MHz, DMSO-*d_6_*) δ 0.89, (s, 3H), 1.02 (s, 3H), 2.01 (d, 1H, *J* =16 Hz), 2.19 (d, 1H, *J* =16 Hz),2.25 (s, 3H) 2.27 (d, 1H, *J* = 12 Hz), 2.40(d, 1H, *J* = 20 Hz), 5.10 (s, 1H), 7.11 (s, 4H), 7.72 (s, NH), 9.43 (s, NH) ppm.


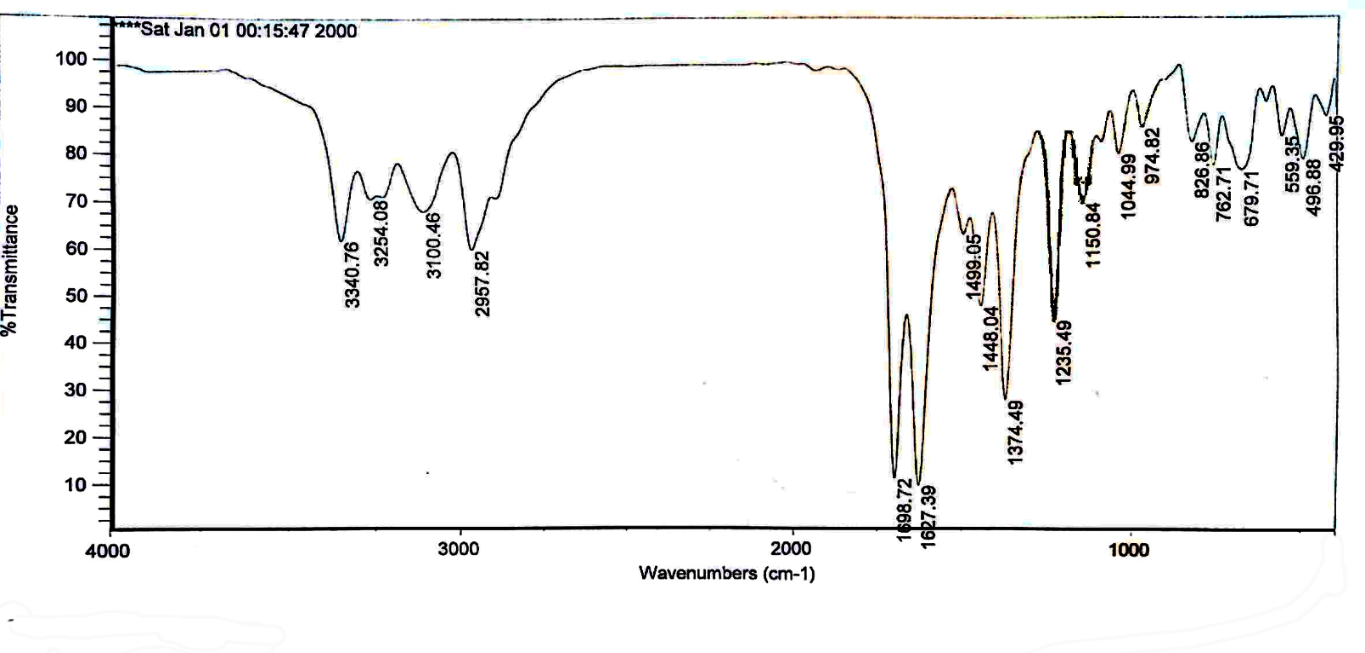


Figure S22: FT-IR spectrum of compound 6f

Figure S23: ^1^H NMR spectrum of compound 6f

**7,7-dimethyl-4-(2,4-**[**dichloro**](https://www.google.com/search?client=firefox-b-e&sca_esv=34f5e017178ef90e&sxsrf=ACQVn0-hEcL8SClQnGPu8-kQbNYnejn1LA:1710906407070&q=2,4-dichloro&spell=1&sa=X&ved=2ahUKEwiVoIyF94GFAxUHQEEAHRgVARYQBSgAegQIChAC)**phenyl)-3,4,7,8-tetrahydroquinazoline-2,5(1H,6H)-dione** (6k)

White solid; IR (KBr) ν (cm^−1^): 3318 (N-H), 3220 (N-H), 3105 (C-H Aromatic), 2961 (C-H Aliphatic), 1702 (C=O), 1626 (C=C), 1508 (C=C), 1236 (C-N). ^1^H NMR (400 MHz, DMSO-*d_6_*) δ 0.95 (s, 3H), 1.02 (s, 3H), 1.98 (d, 1H, *J* =16 Hz), 2.16 (d, 1H, *J*=16Hz), 2.32 (d, 1H, *J* = 20 z), 2.43 (d, 1H, *J* = 20 Hz), 5.53(s, 1H), 7.29 (d, 1H, *J* = 8 Hz), 7.40 (d, 1H, *J* = 8 Hz) 7.54 (s, 1H), 7.75 (s, NH), 9.58 (s, NH) ppm.


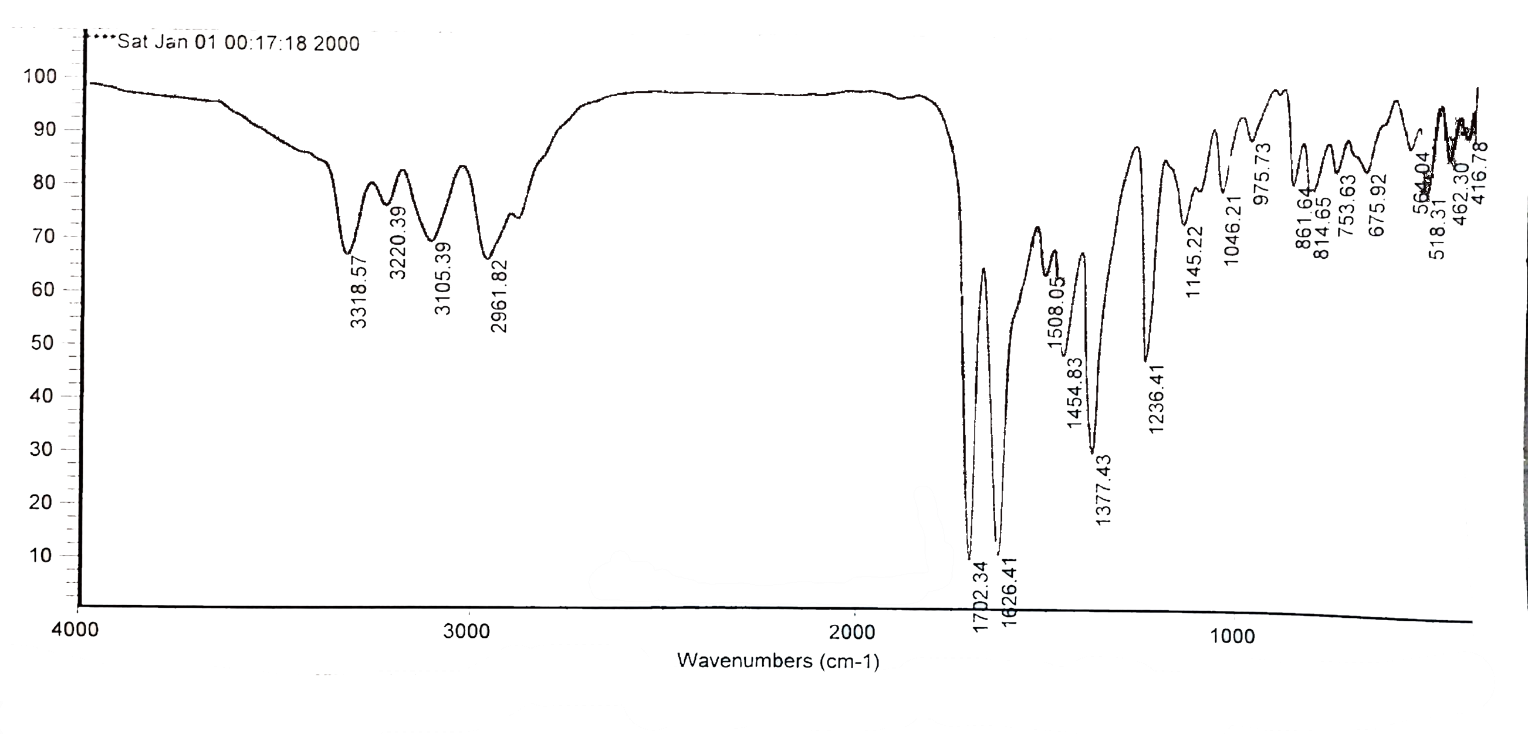


Figure S24: FT-IR spectrum of compound 6k

Figure S25: ^1^H NMR spectrum of compound 6k
